# Smoking and inequalities in mortality in 11 European countries: a birth cohort analysis

**DOI:** 10.1186/s12963-021-00247-2

**Published:** 2021-01-30

**Authors:** Di Long, Johan Mackenbach, Pekka Martikainen, Olle Lundberg, Henrik Brønnum-Hansen, Matthias Bopp, Giuseppe Costa, Katalin Kovács, Mall Leinsalu, Maica Rodríguez-Sanz, Gwenn Menvielle, Wilma Nusselder

**Affiliations:** 1 Department of Public Health, Erasmus MC, P.O. Box 2040, 3000 CA Rotterdam, The Netherlands; 2 Population Research Unit, Faculty of Social Sciences, University of Helsinki, Helsinki, Finland; 3 Department of Public Health Sciences, Stockholm University, Stockholm, Sweden; 4 Department of Public Health, University of Copenhagen, Copenhagen, Denmark; 5 Epidemiology, Biostatistics and Prevention Institute, University of Zurich, Zurich, Switzerland; 6 Department of Clinical Medicine and Biology, University of Turin, Torino, Italy; 7 Demographic Research Institute, Budapest, Hungary; 8 Stockholm Centre for Health and Social Change, Södertörn University, Huddinge, Sweden; 9 Department of Epidemiology and Biostatistics, National Institute for Health Development, Tallinn, Estonia; 10 Agència de Salut Pública de Barcelona, Barcelona, Spain; 11 CIBER Epidemiología y Salud Pública (CIBERESP), Madrid, Spain; 12 Sorbonne Université, INSERM, Institut Pierre Louis d’Epidémiologie et de Santé Publique, Paris, France

**Keywords:** Birth cohort, Smoking, Mortality, Educational inequalities

## Abstract

**Purpose:**

To study the trends of smoking-attributable mortality among the low and high educated in consecutive birth cohorts in 11 European countries.

**Methods:**

Register-based mortality data were collected among adults aged 30 to 79 years in 11 European countries between 1971 and 2012. Smoking-attributable deaths were estimated indirectly from lung cancer mortality rates using the Preston-Glei-Wilmoth method. Rate ratios and rate differences among the low and high-educated were estimated and used to estimate the contribution of inequality in smoking-attributable mortality to inequality in total mortality.

**Results:**

In most countries, smoking-attributable mortality decreased in consecutive birth cohorts born between 1906 and 1961 among low- and high-educated men and high-educated women, but not among low-educated women among whom it increased. Relative educational inequalities in smoking-attributable mortality increased among both men and women with no signs of turning points. Absolute inequalities were stable among men but slightly increased among women. The contribution of inequality in smoking-attributable mortality to inequality in total mortality decreased in consecutive generations among men but increased among women.

**Conclusions:**

Smoking might become less important as a driver of inequalities in total mortality among men in the future. However, among women, smoking threatens to further widen inequalities in total mortality.

**Supplementary Information:**

The online version contains supplementary material available at 10.1186/s12963-021-00247-2.

## Introduction

While people’s health in Europe has improved in the last few decades, the improvements are not experienced equally by all. Reducing educational inequalities in health has been one of the most important targets in public health [1]. Among all health risks, smoking is believed to be the greatest avoidable risk factor [2] and one of the leading contributors to inequalities in mortality [3–6]. In Europe, smoking prevalence has declined in many countries [7], partly as a result of tobacco control policies [8, 9].

However, it has been less studied how changes in smoking behavior have influenced trends in educational inequalities in mortality. The studies that measured the effect of smoking on inequalities in mortality have suggested increases in relative inequalities in smoking-related mortality in many European countries in recent decades [10–13]. Although with different methods, these studies all used a period approach. However, other studies have established that the evolution of the smoking epidemic and lung cancer epidemic are phenomena related to birth cohorts [14, 15]. The habit of smoking is adopted in the early stages of life and is likely to persist throughout the life course [16]. The likelihood of picking up smoking varies over time, due to historical differences in the physical or social environment, such as access to cigarettes, socioeconomic conditions, or social acceptance of smoking in time of uptake smoking [17, 18]. These effects are likely to be only partially captured by period effects. Therefore, a birth cohort perspective can offer us a more comprehensive understanding not only of the evolution of mortality due to smoking in the population as a whole but also of educational inequalities in mortality from smoking. It may also offer better insights into how inequalities in mortality are likely to evolve in the future.

In this article, we studied trends in smoking-attributable mortality among the low and high-educated in consecutive birth cohorts in 11 European countries. We assessed whether educational inequalities in smoking-attributable mortality and their contribution to educational inequalities in total mortality have changed between older and younger generations in these countries. We used the Preston-Glei-Wilmoth (PGW) method to estimate smoking-attributable mortality.

## Data and methods

### Data

Register-based mortality data were collected and harmonized for all-cause mortality and lung cancer mortality by sex, age (5-year age group from 30 to 34 to 75-79 years, except Norway for which only 10-year age groups were available for the ages 30-49), and educational level over the period 1971-2012 in 11 European countries (Table 1). For those of age 30-39 and 40-49 in Norway, we estimated the mortality number in 5-year age groups using the Human Mortality Database (www.mortality.org). Most data comprise national populations, except for Italy, where we obtained data from Turin only; for Spain, from Barcelona only; and for the UK, from England and Wales only. Details on data sources can be found in Supplementary file 1.

**Table 1:** Characteristics of the mortality data and population data used in the analyses in each country: Types of data set (longitudinal data or cross-sectional data: unlinked or repeated); geographic coverage of the dataset (National or Urben); the period covered in the dataset; the number of birth cohorts constructed based on the dataset; the oldest birth cohort constructed; the youngest birth cohort constructed; person-years at risk followed up; number of deaths due to lung cancer; and the percentage of lung cancer deaths with low education in each gender

Population	Type of dataset^1^	Geographic coverage	Period	Number of birth cohorts	Oldest birth cohort	Youngest birth cohort	P-Y^2^ follow-up	Number of deaths^3^	% of lung cancer deaths with low education	
									Male	Female
Belgium	Longitudinal	National	1991-2006	6	1927-1936	1952-1961	78819	77795	77.8	74.3
Denmark	Longitudinal	National	1991-2010	6	1926-1934	1951-1959	47377	53491	47.2	63.4
Estonia	CS, unlinked	National	1987-2012	5	1928-1936	1948-1956	9551	11715	59.0	47.4
Finland	Longitudinal	National	1971-2011	11	1902-1910	1952-1960	84266	71774	81.0	74.1
Hungary	CS, unlinked	National	1971-2010	9	1909-1916	1949-1956	101767	106780	83.9	76.9
Italy (Turin)	Longitudinal	Urban	1971-2011	8	1907-1916	1942-1951	13983	17722	85.7	91.5
Lithuania	CS, unlinked	National	1988-2011	5	1932-1940	1952-1960	16449	14428	56.6	45.3
Norway	Longitudinal	National	1971-2006	9	1902-1910	1942-1950	53347	44959	55.6	61.1
Spain (Barcelona)	CS, repeated	Urban	1992-2011	7	1923-1931	1953-1961	13723	14313	72.4	75.2
Sweden	Longitudinal	National	1990-2005	6	1921-1929	1946-1954	66980	39996	56.5	54.5
Switzerland	Longitudinal	National	1991-2010	7	1922-1930	1952-1960	59446	43679	33.8	49.1

^1^CS represents cross-sectional

^2^P-Y represents person-years. Person-years at risk is given in thousands

^3^Number of deaths refers to deaths due to lung cancer

Educational level was measured as the highest level achieved and coded into three groups based on the International Standard Classification of Education (ISCED-97): Up to lower secondary education (ISCED 0, 1 and 2; “low”), completed upper secondary education (ISCED 3 and 4; “mid”) and tertiary education (ISCED 5 and 6; “high”). In the UK (England and Wales), only two groups could be distinguished, namely the “low” (ISCED 0, 1, 2, and 3) and “high” (ISCED 5 and 6).

Birth cohorts were reconstructed using age and period (*birth cohort* = *period* − *age*) in the mortality dataset by country, sex, and level of education. Because data were collected in 5-year age groups and 1- to 5-year periods, the birth cohorts reconstructed were in 6- to 10-year ranges with overlaps. For example, those of age 30 to 34 who died during 1980-1984 belong to birth cohort 1946-1954, and those of age 35 to 39 who died during the same period belong to birth cohort 1941-1949. In each country-sex-education level-age-period group, we excluded cells in which the number of deaths due to lung cancer was less than 10 in order to avoid erratic results due to small numbers. We further excluded birth cohorts with data for less than three age groups. As a result, our analysis covers 18 birth cohort groups, ranging from 1902-1910 to 1952-1961. We present birth cohorts by their median birth year. Detailed descriptions of the birth cohort construction are provided in Supplementary file 2_a and 2_b.

### Methods

The method consisted of three steps. The first step used the PGW method to estimate the period- and age-specific number of deaths attributable to smoking, by country, sex, and level of education, from the number of lung cancer deaths. Total numbers of deaths in the same groups were also obtained for later use. The second step performed a form of direct standardization method yielding comparative cohort mortality figures (CCMF) to summarize overall mortality from smoking in each birth cohort. The final step calculated rate ratios (RR), rate differences (RD), and contributions of inequalities in smoking-attributable mortality to educational inequalities in total mortality. In the following, we describe each of these steps in more detail.

#### The PGW method

The PGW method, introduced by Preston et al. [19], is based on a regression analysis which uses the lung cancer death rate to estimate smoking-attributable mortality from all causes, and was originally performed on data of people aged 50 and higher in 21 high-income countries from 1950 to 2007. For the age group 30-49, we used an extended PGW model developed by Martikainen et al. [20]. We applied this method to each country-sex-education level-cohort group in our data.

First, we compared lung cancer death rates (*M*) in our study to lung cancer death rates among non-smokers (*λ*) in the Second Prospective Cancer Prevention Study (CPS-II) to get the proportion of lung cancer deaths attributable to smoking (${A}_L=\frac{M-\lambda }{M}$). Second, we calculated the proportion of other deaths attributable to smoking (${A}_O=1-{e}^{-{\beta}^{\prime}\left(M-\lambda \right)}$). The parameters (*β*^′^) were published in the original articles and can be directly used without re-performing the analysis in further studies to obtain the proportion of other deaths. Finally, we combined the two fractions calculated above, weighted by the numbers of deaths from lung cancer (*D*_*L*_) and other causes (*D*_*O*_), to generate the overall smoking-attributable fraction for deaths from all causes ($A=\frac{A_L{D}_L+{A}_O{D}_O}{D_L+{D}_O}$). We obtained the overall number of deaths due to smoking by multiplying the overall smoking-attributable fraction to the number of total deaths. We calculated smoking-attributable death rates using the overall number of deaths due to smoking and person-years in each country-sex-education level-cohort group. Details of calculation can be found in Supplementary file 3.

#### The CCMF method

We used the CCMF method, introduced by Gardner et al. [21], to compare mortality between birth cohorts that have different age compositions. The CCMF method is a form of direct standardization and is described in Supplementary file 4. We summed the person-years in all countries by sex and age to obtain a standard population. Briefly, the resulting CCMF is the ratio of the expected deaths in a birth cohort if its age-specific observed mortality rates would apply to the standard population in the same age groups, to the observed deaths in the standard population in the same cohort.

$$ CCMF=\sum \limits_i\frac{\mathrm{Expected}\ \mathrm{deaths}\ \mathrm{in}\ \mathrm{standard}\ \mathrm{population}\ \mathrm{at}\ \mathrm{cohort}\ \mathrm{rates}}{\mathrm{Observed}\ \mathrm{deaths}\ \mathrm{in}\ \mathrm{standard}\ \mathrm{population}\ \mathrm{at}\ \mathrm{standard}\ \mathrm{rates}}=\sum \limits_i\frac{Y_{si}\ {r}_{cseki}}{D_{si}} $$

$$ =\sum \limits_i\frac{Y_{si}\ {r}_{cseki}}{Y_{si}\ {R}_{si}}=\sum \limits_i\frac{r_{cseki}}{R_{si}} $$

where *i* = age group, *s* = sex, *c* = country, *e* = level of education, *k* = birth cohort,

*Y*_*si*_ = standard population in gender *s* and age group *i*

*r*_*cseki*_ = observed death rate in country *c*, gender *s*, education level *e*, cohort *k*, and age group *i*

*D*_*si*_ = number of deaths in standard population in gender *s* and age group *i*

*R*_*si*_ = standard death rate in standard population in gender *s* and age group *i*

#### Rate ratios, rated differences, and the contributions

To estimate relative inequalities in mortality, we calculated RR, by taking the ratio between the CCMFs of the low-educated and the CCMFs of the high-educated in each birth cohort by sex and country. To estimate absolute inequalities, we calculated RD, for which we first calculated smoking-attributable mortality rates by sex in the standard population. Next, we multiplied these standard smoking-attributable mortality rates with the CCMF of every birth cohort to obtain the absolute smoking-attributable mortality rates for every birth cohort in each country-sex-education group. RDs were then calculated by subtraction of the smoking-attributable mortality rate of the high educated from that of the low educated. In the same manner, we calculated RDs of the total mortality rates, which we used to obtain the fraction of smoking-attributable mortality from inequalities in total mortality.

We repeated our analyses using less strict exclusion criteria, which also included birth cohorts with data for only two age groups. We present the results in Supplementary file 5.

We calculated 95% confidence intervals (95% CI) using parametric bootstrapping, assuming Poisson-distributed death counts and setting the number of repetitions to 1000. All analyses were performed using Stata V.15.1 SE.

## Results

### Smoking-attributable mortality

**Fig. 1:**
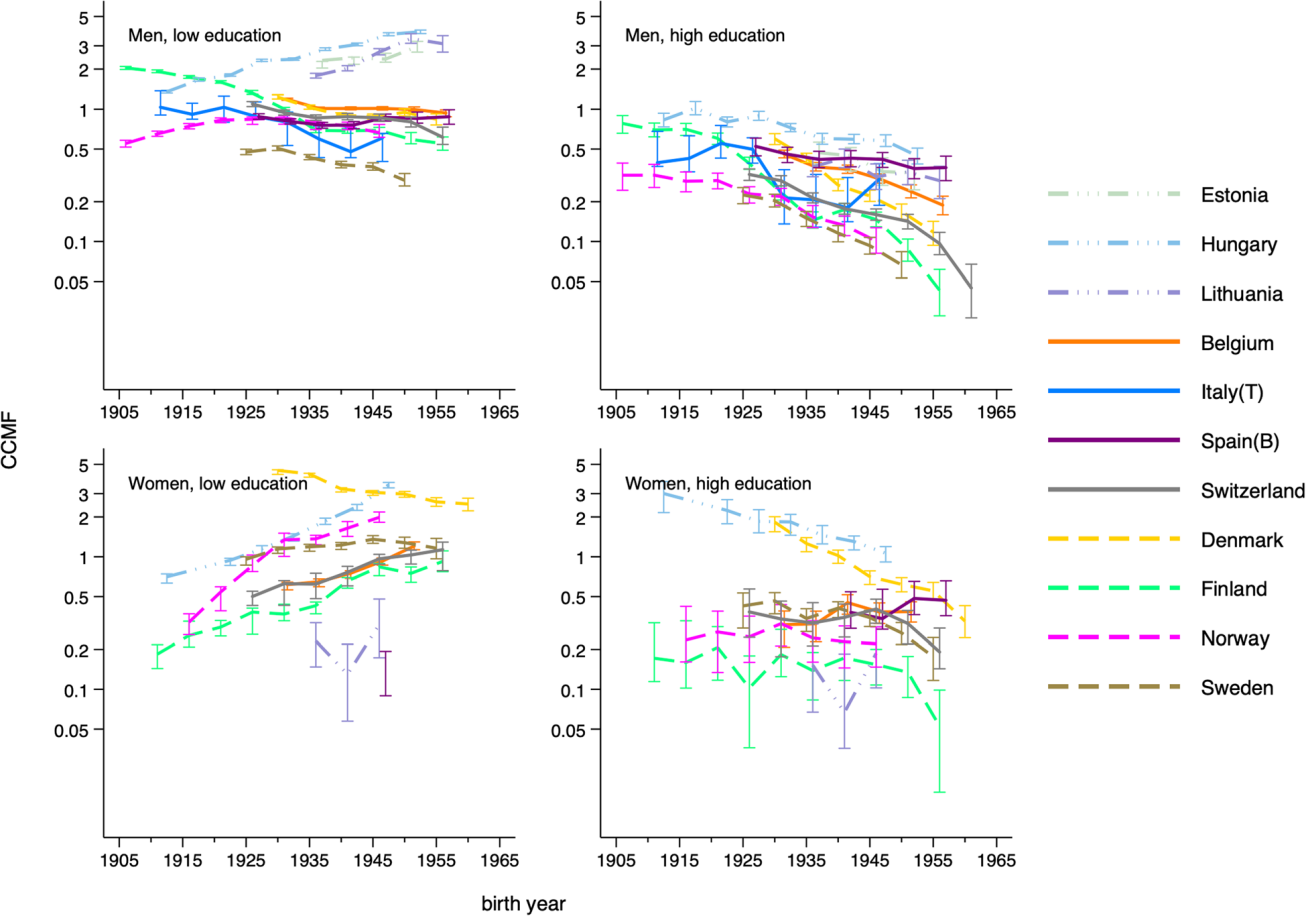
Birth cohort trends in age-standardized smoking-attributable mortality in 11 European countries, by education and gender

Table 1 shows the characteristics of the mortality and population data used in the analyses. Figure 1 presents birth-cohort trends in age-standardized smoking-attributable mortality in 11 countries, by education and gender. When a CCMF value is higher than 1, the age-standardized smoking-attributable mortality for that birth cohort is higher than the average age-standardized smoking-attributable mortality of 11 countries in the same sex group.

For men, smoking-attributable mortality was higher among the low educated than among the high educated in every country. Smoking-attributable mortality gradually decreased in consecutive birth cohorts among both low and high-educated men in most countries, except for low-educated men in Estonia and Hungary, where smoking-attributable mortality increased, and in Lithuania, where a decrease followed after an increase. Among women, smoking-attributable mortality was not always higher in the low educated than the high educated. Among high-educated women, smoking-attributable mortality decreased in consecutive generations in most countries. Among low-educated women, however, smoking-attributable mortality gradually increased in consecutive cohorts in most countries, except for Denmark where it declined from older generations at a very high level.

### Relative and absolute inequalities in smoking-attributable mortality

**Fig. 2:**
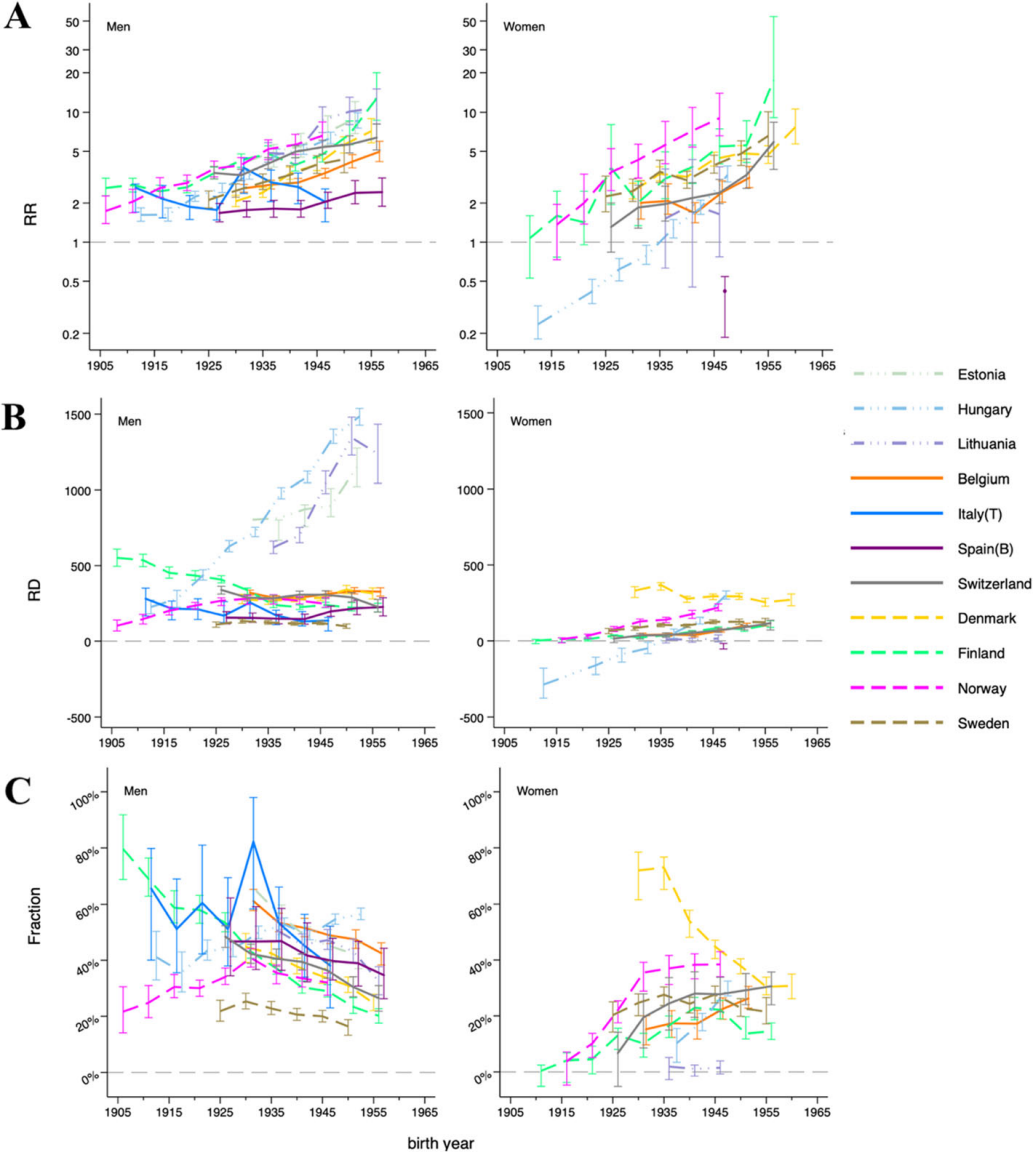
Relative inequalities (**a**), absolute inequalities (**b**) in smoking-attributable mortality, the contribution of smoking to absolute inequalities in total mortality (**c**), in 11 European countries by sex and by educational leve. (We excluded generations born before 1936 in Hungary because they had higher smoking-attributable mortality among the high educated and higher total mortality rates among the low educated in older generations).

Relative inequalities were present among men in all generations in all countries (Fig. 2a, Supplementary file 7). Relative inequalities increased greatly in consecutive birth cohorts except for Italy where relative inequalities reached a peak among the generation born around 1931, and declined in the younger generations. Among women, relative inequalities were present among all generations in most countries, but reverse inequalities, with higher smoking-attributable mortality among the high-educated than among the low-educated, were present among the generations born before 1936 in Hungary. Compared to men, relative inequalities increased more dramatically among women in consecutive birth cohorts: they started with smaller relative inequalities in older generations but reached larger inequalities in the younger generations.

Absolute inequalities (Fig. 2b, Supplementary file 7) were stable in consecutive birth cohorts in many countries, except in Hungary, Estonia, and Lithuania where absolute inequalities increased largely, and in Finland and Italy where absolute inequality decreased slightly in consecutive generations. Among women, absolute inequalities increased subtly in consecutive birth cohorts in most countries and increased largely in Hungary where reverse inequalities were present for those born before 1936.

### Contribution of smoking to inequalities in total mortality

Absolute inequalities in total mortality (Supplementary file 6) increased in consecutive generations in all countries among both men and women. Figure 2c (Supplementary file 7) shows the extent to which inequalities in smoking-attributable mortality contributed to these inequalities in total mortality in consecutive generations. Among men, the contributions declined in consecutive birth cohorts in most countries. In Norway, the contribution reached a peak among the generation born around 1931 and declined in younger generations. In Hungary, the contributions increased over consecutive generations. Among women, the contributions increased in consecutive birth cohorts in many countries, except for Denmark where it reached a peak with the generation born around 1936, followed by a decline.

Overall, we are confident in most of our results except for RR for the youngest generation of women in Finland and the contribution for men in Italy, where the range of 95% CIs are excessively large.

## Discussion

### Main findings

We found that in most countries, smoking-attributable mortality decreased in consecutive birth cohorts among low- and high-educated men and high-educated women, but not among low-educated women in whom it increased. In terms of educational inequalities in smoking-attributable mortality, we found steep increases in relative inequalities among both men and women, stable absolute inequalities among men and subtle increases in absolute inequalities among women in consecutive generations in most countries. The contribution of inequalities in smoking-attributable mortality to inequalities in total mortality decreased among men and increased among women in consecutive generations in most countries. The decreasing trends in the contribution of inequalities among men are the result of stable absolute inequalities in smoking-attributable mortality and larger increases of absolute inequalities in total mortality; the increasing trends among women are the results of larger increases of absolute inequalities in smoking-attributable mortality and smaller increases of absolute inequalities in total mortality.

### Limitations and strengths

Our study applies the innovative PGW method, which uses lung cancer deaths to estimate the overall number of deaths due to smoking. Essentially, this method assumes that there is a constant relationship between lung cancer mortality and smoking-related mortality from other causes of death. It is based on the CPS-II studies which provide an estimate of lung cancer mortality among non-smokers in the USA [22], but whether the same rates apply elsewhere is unknown. The PGW method may overestimate the damage from smoking in countries with unusually low mortality from other causes. This might be the case in some Mediterranean countries where cardiovascular disease mortality has much less impact on total mortality [23]. The PGW method may misjudge the impact of smoking with unusually high mortality from other causes. High mortality rates in middle age in central European countries were observed in our data, where middle-aged people may have died from other causes before potentially developing lung cancer. Therefore, the proportion of lung cancer deaths attributable to smoking may have been underestimated. However, it is noteworthy that we used the coefficients presented in the Preston et al. paper, and that regression analyses were based on several European countries including Eastern European countries. The PGW method further assumes the overall damage caused by the mix of prevalence, duration, and intensity of smoking based on lung cancer mortality is proportionally the same for other causes. This assumption may be unreasonable. However, it is very difficult to measure these factors, let alone estimate their damage to deaths [24]. In our application of the PGW method, we also assume that the relationship between lung cancer mortality and smoking-related mortality from other causes is the same in all education groups. This may not be true. For example, low-educated non-smokers might suffer from a higher risk of lung cancer than high-educated non-smokers due to other exposures [25], which could have contributed to an overestimation of smoking-attributable mortality among the low educated. The PGW method also does not distinguish between exposure and susceptibility to smoking, both of which may vary by level of education. Although several studies have assessed some of these issues and found that the PGW provides robust results [13, 26, 27], we recommend replication of our findings with other methods, e.g., survey-based smoking prevalence data by level of education analyzed in a cohort perspective.

Our study has several other limitations. First, it is a simple descriptive analysis, without a formal age-period-cohort analysis, which does not allow us to quantify the extent of the cohort-related changes, as compared to period- and age-related changes in mortality. Although our results are highly suggestive of cohort effects, it will be interesting to see this confirmed in a formal age-period-cohort analysis [28, 29].

Second, despite the large size of our dataset, the scope of our analyses was sometimes restricted by small number problems. Due to the slower evolution of higher educational attainment among women, especially in Mediterranean countries, we could not always closely follow the evolution of inequalities in smoking-attributable mortality among women. Moreover, to calculate age-standardized smoking-attributable mortality and inequalities for different birth cohorts, we stratified mortality by sex, level of education, age, and period, causing small numbers of deaths in some cells. We had to apply strict exclusion criteria to avoid erratic results due to these small numbers, which reduced the number of birth cohorts and countries included in the analysis. In a sensitivity analysis, we applied less strict exclusion criteria, which allowed us to include more birth cohorts, both early and recent generations and more countries. However, the results remain largely unchanged in this extended analysis (Supplementary file 5).

Third, the constructed birth cohorts do not contain the same age groups. We used the CCMF to maximize comparability between birth cohorts that consist of different age groups. In this method, we compared mortality in each cohort with the standard population of the same age range. This may have introduced bias in the comparison of relative inequality between birth cohorts of different age ranges because relative inequalities in mortality tend to be larger at younger ages [30]. The CCMF method does not fully adjust for these differences in age structure.

Finally, the coverages of data were different between countries. Italy and Spain did not offer national-level datasets, but mortality patterns have been shown to differ little between these (large) urban regions compared with the national average [31, 32]. Moreover, there were only two levels of education in the data from the UK, resulting in some mid-educated (ISCED 3) populations categorized as low-educated. This might have caused underestimation of the inequalities of the UK.

The strengths of our study include using a unique set of data on mortality by education, covering a wide range of time periods and countries. Most mortality data were based on individual mortality follow up, avoiding numerator-denominator bias [33]. The approach of the PGW method avoids several limitations with the population attributable fraction (PAF) method, which is based on the prevalence of self-reported smoking and relative risks. Firstly, there are possible differences in self-reports between different surveys [34], between different education groups [35], and between different countries. Secondly, currently available relative risks of smoking-related mortality are not specific by countries and level of education. Using general relative risks may over(under)estimate smoking attributed mortality if the proportion of non-smoking-related causes in total mortality is high (low) in that specific country [36]. Third, the prevalence and relative risk used in the PAF approach generally do not account for the impact of duration and intensity of smoking on mortality. A final strength is that we focused on birth-cohort trends in smoking-attributable mortality by education and birth-cohort trends in inequalities. The birth cohort perspective enables us to estimate the damage by smoking cumulated over the life-course and can provide useful insights into the impact of smoking on future inequalities in mortality.

### Interpretation

The trends in smoking-attributable mortality in consecutive generations by educational level for men and women were, to some extent, consistent with the updated cigarette epidemic model, which shows that smoking-attributable mortality has decreased in men and increased or plateaued in women in the last decades of the twentieth century [37]. Low-educated women were a few generations behind low-educated men in the cigarette epidemic: when generations of low-educated women still showed increases of smoking-attributable mortality, the same generations of low-educated men have already reached the decline stage. Exceptions were in Denmark, where low-educated women already reached the stage of decline in the generation born after the first quarter of the twentieth century.

Several studies have examined the trends of smoking-related mortality in European countries by birth cohort and sex using age-period-cohort models [14, 38–40]. These studies showed a prominent role of cohort effects in shaping smoking-related mortality trends, together with little evidence for period effects. These studies showed decreasing trends in smoking-attributable mortality in consecutive cohorts among men and increasing trends among women in most countries but did not differentiate by level of education. Our results show similar trends for low-educated men in most countries except for Denmark, Estonia, Lithuania, and Spain, and among low-educated women in most countries except for Denmark and Sweden. Our results of high-educated men in some countries and high-educated women in most counties are not in line with previous studies, showing that population averages may hide important educational differences.

Gregoraci et al. [13] compared inequalities in smoking-attributable mortality between the low and high educated in 14 European countries in two time periods 2000-2004 and 1990-1994, using the PGW-method like we do, but without applying a cohort perspective. They found increases in relative inequalities and decreases in absolute inequalities among men, and increases in both relative and absolute inequalities among women. Our results are based on a longer time period and we found larger relative inequalities among men and women in younger generations and increasing absolute inequalities. The difference between their study and ours may be due to their period perspective that mixes older generations with lower absolute inequalities together with younger generations with higher absolute inequalities.

Several studies examined cohort patterns in smoking initiation and cessation and found rising disparities between groups of low and high educated [18, 41, 42], which lends support to our findings.

## Conclusion

Our study found, in most counties, decreasing smoking-attributable mortality trends in consecutive birth cohorts among both low- and high-educated men and among high-educated women, but increasing smoking-attributable mortality among low-educated women. For men, based on these birth cohort-specific patterns, we expect that in most non-Eastern European countries smoking will become less important as a driver of inequalities in total mortality in the future, despite a continuing widening of relative inequalities in smoking-attributable mortality. Among men in Hungary, Estonia, and Lithuania, smoking may well remain an important driver of inequalities in total mortality in the near future. Among women, however, smoking threatens to further widen inequalities in total mortality. In order to avoid this, it is necessary to step up efforts to promote smoking prevention and cessation while not increasing health inequalities. Before implementation, interventions should be equity-checked and evaluated [43]. Population-level interventions such as price increase to tobacco and sale restriction to minors have the potential to reduce inequalities [44].

## Supplementary Information

**Additional file 1: Supplementary file 1** Overview of mortality data source. **Supplementary file 2**_a. Detailed birth cohorts constructed by country. **Supplementary file 2**_b. Age composition of each birth cohort. **Supplementary file 3**. Calculations of smoking-attributable mortality from the Preston-Glei-Wilmoth method. **Supplementary file 4** Calculations of CCMFs. **Supplementary file 5**. CCMF results from less strict exclusion criteria. **Supplementary file 6**. Birth cohort-specific trends of total mortality. Additional file 8: **Supplementary file 7**. Table for rate ratio, rate difference and the fraction with 95% CI

**Additional file 2.**

## Data Availability

Available upon request.

## Abbreviations

PGW: Preston-glei-wilmoth
ISCED: International standard classification of education
CCMF: Comparative cohort mortality figures
RR: Rate ratio
RD: Rate difference
CPS-II: Second prospective cancer prevention study
CI: Confidence intervals
PAF: Population attributable fraction
P-Y: Person-years
CS: Cross-sectional

## References

[1] M. Marmot et al., “WHO European review of social determinants of health and the health divide,” Lancet, vol. 380, no. 9846, pp. 1011-1029, Sep 15 2012, doi: 10.1016/S0140-6736(12)61228-8. 10.1016/S0140-6736(12)61228-8 22964159

[2] Eurobarometer S. Attitudes of Europeans towards tobacco and electronic cigarettes. TNS Opin Soc. 2015;429:214.

[3] U. D. o. H. a. H. Services, “How tobacco smoke causes disease: what it means to you. Atlanta: Centers for Disease Control and Prevention,” National Center for Chronic Disease Prevention and Health Promotion, Office on Smoking and Health 2010.

[4] Eikemo TA (2014). How can inequalities in mortality be reduced? A quantitative analysis of 6 risk factors in 21 European populations. PLoS One.

[5] J. P. Mackenbach et al., “Socioeconomic inequalities in health in 22 European countries,” N Engl J Med, vol. 358, no. 23, pp. 2468-2481, Jun 5 2008, doi: 10.1056/NEJMsa0707519. 10.1056/NEJMsa0707519 18525043

[6] Ostergren O, Martikainen P, Tarkiainen L, Elstad JI, Bronnum-Hansen H (2019). Contribution of smoking and alcohol consumption to income differences in life expectancy: evidence using Danish, Finnish, Norwegian and Swedish register data. J Epidemiol Community Health.

[7] Islami F, Torre LA, Jemal A (2015). Global trends of lung cancer mortality and smoking prevalence. Transl Lung Cancer Res.

[8] Feliu A (2019). Impact of tobacco control policies on smoking prevalence and quit ratios in 27 European Union countries from 2006 to 2014. Tob Control.

[9] So VH, Best C, Currie D, Haw S (2019). Association between tobacco control policies and current smoking across different occupational groups in the EU between 2009 and 2017. J Epidemiol Community Health.

[10] P. Jha, R. Peto, W. Zatonski, J. Boreham, M. J. Jarvis, and A. D. Lopez, “Social inequalities in male mortality, and in male mortality from smoking: indirect estimation from national death rates in England and Wales, Poland, and North America,” Lancet, vol. 368, no. 9533, pp. 367-370, Jul 29 2006, doi: 10.1016/S0140-6736(06)68975-7. 10.1016/S0140-6736(06)68975-7 16876664

[11] Stringhini S (2011). Health behaviours, socioeconomic status, and mortality: further analyses of the British Whitehall II and the French GAZEL prospective cohorts. PLoS Med.

[12] M. C. Kulik et al., “Educational inequalities in three smoking-related causes of death in 18 European populations,” (in eng), Nicotine Tob Res, vol. 16, no. 5, pp. 507-518, May 2014, doi: ntt175 [pii]10.1093/ntr/ntt175. 10.1093/ntr/ntt175 24212763

[13] G. Gregoraci et al., “Contribution of smoking to socioeconomic inequalities in mortality: a study of 14 European countries, 1990-2004,” (in eng), Tob Control, vol. 26, no. 3, pp. 260-268, May 2017, doi: tobaccocontrol-2015-052766 [pii]10.1136/tobaccocontrol-2015-052766. 10.1136/tobaccocontrol-2015-052766 27122064

[14] F. I. Bray and E. Weiderpass, “Lung cancer mortality trends in 36 European countries: secular trends and birth cohort patterns by sex and region 1970-2007,” (in eng), Int J Cancer, vol. 126, no. 6, pp. 1454-1466, Mar 15 2010, doi: 10.1002/ijc.24855. 10.1002/ijc.24855 19728330

[15] Pampel FC (2005). Diffusion, cohort change, and social patterns of smoking(). Soc Sci Res.

[16] Troost JP (2012). An updated global picture of cigarette smoking persistence among adults. J Epidemiol Glob Health.

[17] Ahacic K, Parker MG, Thorslund M (2007). Aging in disguise: age, period and cohort effects in mobility and edentulousness over three decades. Eur J Ageing.

[18] S. Legleye, M. Khlat, F. Beck, and P. Peretti-Watel, “Widening inequalities in smoking initiation and cessation patterns: a cohort and gender analysis in France,” Drug Alcohol Depend, vol. 117, no. 2-3, pp. 233-241, Sep 1 2011, doi: 10.1016/j.drugalcdep.2011.02.004. 10.1016/j.drugalcdep.2011.02.004 21420251

[19] S. H. Preston, D. A. Glei, and J. R. Wilmoth, “A new method for estimating smoking-attributable mortality in high-income countries,” (in eng), Int J Epidemiol, vol. 39, no. 2, pp. 430-438, Apr 2010, doi: dyp360 [pii]10.1093/ije/dyp360. 10.1093/ije/dyp360 PMC2915474 20032265

[20] Martikainen P, Makela P, Peltonen R, Myrskyla M (2014). Income differences in life expectancy: the changing contribution of harmful consumption of alcohol and smoking. Epidemiology.

[21] Gardner MJ, Osmond C (1984). Interpretation of time trends in disease rates in the presence of generation effects. Stat Med.

[22] D. R. Shopland, D. M. Burns, and National Cancer Institute (U.S.). Smoking and Tobacco Control Program., Changes in cigarette-related disease risks and their implication for prevention and control (Smoking and tobacco control monograph, no. 8). Bethesda, Md.: National Institutes of Health, National Cancer Institute, 1997, pp. xxxvii, 565 p.

[23] Kromhout D (2001). Epidemiology of cardiovascular diseases in Europe. Public Health Nutr.

[24] M. Wensink, J. A. Alvarez, S. Rizzi, F. Janssen, and R. Lindahl-Jacobsen, “Progression of the smoking epidemic in high-income regions and its effects on male-female survival differences: a cohort-by-age analysis of 17 countries,” BMC Public Health, vol. 20, no. 1, p. 39, Jan 10 2020, doi: 10.1186/s12889-020-8148-4. 10.1186/s12889-020-8148-4 PMC6954612 31924192

[25] Hovanec J (2018). Lung cancer and socioeconomic status in a pooled analysis of case-control studies. PLoS One.

[26] Martikainen P, Ho JY, Preston S, Elo IT (2013). The changing contribution of smoking to educational differences in life expectancy: indirect estimates for Finnish men and women from 1971 to 2010. J Epidemiol Community Health.

[27] L. Blue and A. Fenelon, “Explaining low mortality among US immigrants relative to native-born Americans: the role of smoking,” (in eng), Int J Epidemiol, vol. 40, no. 3, pp. 786-793, Jun 2011, doi: dyr011 [pii]10.1093/ije/dyr011. 10.1093/ije/dyr011 PMC3147070 21324939

[28] Clayton D, Schifflers E (1987). Models for temporal variation in cancer rates. I: Age-period and age-cohort models. Stat Med.

[29] Clayton D, Schifflers E (1987). Models for temporal variation in cancer rates. II: Age-period-cohort models. Stat Med.

[30] M. Huisman et al., “Educational inequalities in cause-specific mortality in middle-aged and older men and women in eight western European populations,” Lancet, vol. 365, no. 9458, pp. 493-500, Feb 5-11 2005, doi: 10.1016/S0140-6736(05)17867-2. 10.1016/S0140-6736(05)17867-2 15705459

[31] Regidor E, Kunst AE, Rodriguez-Artalejo F, Mackenbach JP (2012). Small socio-economic differences in mortality in Spanish older people. Eur J Public Health.

[32] Marinacci C (2013). Social inequalities in total and cause-specific mortality of a sample of the Italian population, from 1999 to 2007. Eur J Public Health.

[33] Valkonen T (1993). Problems in the measurement and international comparisons of socio-economic differences in mortality. Soc Sci Med.

[34] Kulik MC, Eikemo TA, Regidor E, Menvielle G, Mackenbach JP (2014). Does the pattern of educational inequalities in smoking in Western Europe depend on the choice of survey?. Int J Public Health.

[35] Wagenknecht LE, Burke GL, Perkins LL, Haley NJ, Friedman GD (1992). Misclassification of smoking status in the CARDIA study: a comparison of self-report with serum cotinine levels. Am J Public Health.

[36] C. o. P. National Research Council. International differences in mortality at older ages: dimensions and sources. Washington, D.C.: National Academies Press; 2011. 21977541

[37] Thun M, Peto R, Boreham J, Lopez AD (2012). Stages of the cigarette epidemic on entering its second century. Tob Control.

[38] C. La Vecchia, E. Negri, F. Levi, A. Decarli, and P. Boyle, “Cancer mortality in Europe: effects of age, cohort of birth and period of death,”(in eng), Eur J Cancer, vol. 34, no. 1, pp. 118-141, 1998, doi: S0959804997003353 [pii]. 10.1016/s0959-8049(97)00335-3 9624248

[39] J. Franco, S. Perez-Hoyos, and P. Plaza, “Changes in lung-cancer mortality trends in Spain,” Int J Cancer, vol. 97, no. 1, pp. 102-105, Jan 1 2002, doi: 10.1002/ijc.1575. 10.1002/ijc.1575 11774250

[40] K. Ahern, “Hemoglobin’s moving around (to the tune of “Santa Claus is Coming to Town”),” (in eng), Biochem Mol Biol Educ, vol. 35, no. 6, p. 478, 2007, doi: 10.1002/bmb.118. 10.1002/bmb.118 21591150

[41] Federico B, Costa G, Kunst AE (2007). Educational inequalities in initiation, cessation, and prevalence of smoking among 3 Italian birth cohorts. Am J Public Health.

[42] Rohrmann S, Becker N, Kroke A, Boeing H (2003). Trends in cigarette smoking in the German centers of the European Prospective Investigation into Cancer and Nutrition (EPIC): the influence of the educational level. Prev Med.

[43] C. Bambra, “First do no harm: developing interventions that combat addiction without increasing inequalities,” Addiction, vol. 113, no. 5, pp. 787-788, 2018, doi: https://doi.org/10.1111/add.14116. 10.1111/add.14116 29314336

[44] Thomas S (2008). Population tobacco control interventions and their effects on social inequalities in smoking: systematic review. Tob Control.

