# Supplementary material for: Smoking and inequalities in mortality in 11 European countries: a birth cohort analysis
Source: Popul Health Metr. 2021 Jan 30;19:3. doi: 10.1186/s12963-021-00247-2 (PMC7847590; doi:10.1186/s12963-021-00247-2)
Supplement: Supplementary file 1 — Additional file 1: Supplementary file 1 Overview of mortality data source. Supplementary file 2_a. Detailed birth cohorts constructed by country. Supplementary file 2_b. Age composition of each birth cohort. Supplementary file 3. Calculations of smoking-attributable mortality from the Preston-Glei-Wilmoth method. Supplementary file 4 Calculations of CCMFs. Supplementary file 5. CCMF results from less strict exclusion criteria. Supplementary file 6. Birth cohort-specific trends of total mortality. Additional file 8: Supplementary file 7. Table for rate ratio, rate difference and the fraction with 95% CI [file 12963_2021_247_MOESM1_ESM.docx]

Article title: Smoking and inequalities in mortality in 11 European countries: a birth cohort analysis

Journal: European Journal of Epidemiology

Author: Di Long^[[1]](#footnote-1)^, Wilma J. Nusselder^[[2]](#footnote-2)^, Pekke Martikainen^[[3]](#footnote-3)^, Olle Lundberg^[[4]](#footnote-4)^, Henrik Brønnum-Hansen^[[5]](#footnote-5)^, Mattias Bopp^[[6]](#footnote-6)^, Olof Östergren^[[7]](#footnote-7)^, Giuseppe Gianluca Costa^[[8]](#footnote-8)^, Katalin Kovács^[[9]](#footnote-9)^, Mall Leinsalu^[[10]](#footnote-10),^^[[11]](#footnote-11)^, Maica Rodríguez-Sanz^[[12]](#footnote-12),^^[[13]](#footnote-13)^, Ramunė Kalėdienė^[[14]](#footnote-14)^, Johan P. Mackenbach^2^

List of table and figures:

Table 1. overview of mortality data source (supplementary file 1)

Table 2. birth cohort reconstructed by age and period of follow-up (supplementary file 2_a)

Table 3. Lexis diagram of the reconstructed birth cohorts by age and period of follow-up (supplementary file 2_a)

Table 4. Birth cohorts reconstructed for each country (supplementary file 2_a)

Table 5. Age composition of each birth cohort (supplementary file 2_b)

Table 6. exact number of rate ratio, rate difference, and fraction of inequality in smoking-attributable mortality to inequality in total mortality, with 95% CI, in each country (supplementary file 7)

Figure 1. Comparative cohort mortality figure of smoking-attributable mortality plotted vs the (median) year of birth. (supplementary file 5)

Figure 2. Relative inequalities (A), absolute inequalities (B) in smoking-attributable mortality, contribution of smoking to absolute inequalities in total mortality (C)*, in 11 European countries by sex and by educational level. (supplementary file 5)

Figure 3. Comparative cohort mortality figure of total mortality plotted vs the (median) year of birth. (supplementary file 6)

Figure 4. Rate ratios and rate difference (per 100,000 person-years) between the low- and the high-educated. (supplementary file 6)

# Supplementary file 1. Overview of mortality data source

Table 1. overview of mortality data source

| Country | Type | Years | Census date | Length of follow-up (year) | Geographic coverage | Inclusion |
| --- | --- | --- | --- | --- | --- | --- |
| Belgium | Longitudinal deaths | 1 March 1990 – Dec 31, 1997 | 1March 1997 | 6yr, 10m | National | All population groups |
|  |  | 1 Oct 2001 – 1 Oct 2006 | 19 Jan 2001 | 10 | National | All population groups |
|  |  | 1 Oct 2006 – 31 Dec 2011 |  |  |  |  |
| Denmark | Longitudinal | 1 Jan 1995 – 31 Dec 1999 | 1995 | 10 | National | All population groups |
|  |  | 1 Jan 2000 – 31 Dec 2004 | 2000 |  | National | All population groups |
|  |  | 1 Jan 2005 – 31 Dec 2009 | 2005 |  | National | All population groups |
|  |  | 1 Jan 2010 – 31 Dec 2014 | 2010 | 10 | National | All population groups |
|  |  | 2011-2014 | 2011 | 4 | National | All population groups |
| Estonia | Longitudinal | 2001-2011 | 31 March 2001 | 11 yr, 9m | National | All population groups |
|  |  | 2012-2015 | Dec 31 2011 | 4 | National | All population groups |
| Finland | Longitudinal | 31 Dec 1970 – 31 Dec 1980 | 31 Dec 1970 | 10 | National | All population groups |
|  |  | 31 Dec 1980 – 31 Dec 1990 | 31 Dec 1980 | 10 | National | All population groups |
|  |  | 31 Dec 1990 – 31 Dec 2000 | 31 Dec 1990 | 10 | National | All population groups |
|  |  | 31 Dec 2000 – 31 Dec 2010 | 31 Dec 2000 | 10 | National | All population groups |
|  |  | 31 Dec 2010 – 31 Dec 2014 | 31 Dec 2010 | 4 | National | 80% representative sample |
| Hungary | Cross-sectional, unlinked | 1971 – 1974 | 1 Jan 1973 | 4 | National | All population groups |
|  |  | 1978 – 1981 | 1 Jan 1980 | 4 | National | All population groups |
|  |  | 1988 – 1991 | 1990 | 4 | National | All population groups |
|  |  | 1999 – 2002 | 2001 | 4 | National | All population groups |
|  |  | 2010-2012 | 2011 | 3 | National | All population groups |
| Italy, Turin | Longitudinal | 24 Oct 1971 – 24 Oct 1981 | 24 Oct 1971 | 10 | City | All population groups |
|  |  | 25 Oct 1981 – 19 Oct 1991 | 25 Oct 1981 | 10 | City | All population groups |
|  |  | 20 Oct 1991 – 20 Oct 2001 | 20 Oct 1991 | 10 | City | All population groups |
|  |  | 21 Oct 2001 – 31 Dec 2010 | 21 Oct 2001 | 9.2 | City | All population groups |
|  |  | 21 Oct 2001 – 9 Oct 2011 | 21 Oct 2001 | 10 | City | All population groups |
|  |  | 10 Oct 2011 – 12 Dec 2013 | 10 Oct 2011 | 2, 3 | City | All population groups |
| Lithuania | Longitudinal^1^ | 6 Apr 2001 – 12 Dec 2009 | 6 April 2001 | 8.8 | National | All population groups |
|  | Longitudinal | 1 March 2011-31 Dec 2014 | 1 March 2011 | 4 | National | All population groups |
| Norway | Longitudinal | Nov 1970 – Dec 1980 | Nov 1970 | 10 | National | All population groups |
|  |  | Nov 1980 – Dec 1990 | Nov 1980 | 10 | National | All population groups |
|  |  | Nov 1990 – Dec 2001 | Nov 1990 | 11 | National | All population groups |
|  |  | Nov 2001 – Dec 2009 | Nov 2001 | 8 | National | All population groups |
| Spain, Barcelona^1^ | Cross-sectional, repeated | 1992-1996 | 1992-1996 | 5 | City | All population groups |
|  |  | 1997-2001 | 1997-2001 | 5 | City | All population groups |
|  |  | 2002-2006 | 2002-2006 | 5 | City | All population groups |
|  |  | 2007-2010 | 2007-2010 | 4 | City | All population groups |
|  |  | 2011-2013 |  | 3 | City | All population groups |
| Sweden | Longitudinal | Jan 1990 – Dec 1999 | Jan 1990 | 10 | National | All population groups |
|  |  | Jan 2000 – Dec 2008 | Jan 2001 | 9 | National | All population groups |
| Switzerland | Longitudinal | Dec 4, 1990 – Dec 5, 2000 | 4.12.1990 | 10 | National | Swiss nationals |
|  |  | Jan 1, 2001 – Dec 31, 2010 | 5.12.2000 | 10 | National | Swiss nationals |
|  |  | Jan 1, 2011 – Dec 31, 2014 | 31.12.2010 | 4 | National | Swiss nationals |

# Supplementary file 2_a. Detailed birth cohorts constructed by country

**The first step is the reconstruction of birth cohort, take Austria as an example.**

Birth cohort is reconstructed as $birth cohort=period-age group$ (Table 1), the Lexis diagram is presented in table 3.

Table 2. birth cohort reconstructed by age and period of follow-up.

| Period of mortality | Age | | | | | | | | | |
| --- | --- | --- | --- | --- | --- | --- | --- | --- | --- | --- |
|  | 75-79 | 70-74 | 65-69 | 60-64 | 55-59 | 50-54 | 45-49 | 40-44 | 35-39 | 30-34 |
| 1991-1996 | 1912-1921 | 1917-1926 | 1922-1931 | 1927-1936 | 1932-1941 | 1937-1946 | 1942-1951 | 1947-1956 | 1952-1961 | 1957-1966 |
|  |  |  |  |  |  |  |  |  |  |  |
| 2001-2006 | 1922-1931 | 1927-1936 | 1932-1941 | 1937-1946 | 1942-1951 | 1947-1956 | 1952-1961 | 1957-1966 | 1962-1971 | 1967-1976 |
|  |  |  |  |  |  |  |  |  |  |  |
| 2006-2011 | 1927-1936 | 1932-1941 | 1937-1946 | 1942-1951 | 1947-1956 | 1952-1961 | 1957-1966 | 1962-1971 | 1967-1976 | 1972-1981 |

Table 3. Lexis diagram of the reconstructed birth cohorts by age and period of follow-up.

| Birth cohort | Age | | | | | | | | | |
| --- | --- | --- | --- | --- | --- | --- | --- | --- | --- | --- |
|  | 30-34 | 35-39 | 40-44 | 45-49 | 50-54 | 55-59 | 60-64 | 65-69 | 70-74 | 75-79 |
| 1912-1921 |  |  |  |  |  |  |  |  |  | 1991-1996 |
| 1917-1926 |  |  |  |  |  |  |  |  | 1991-1996 |  |
| 1922-1931 |  |  |  |  |  |  |  | 1991-1996 |  | 2001-2006 |
| 1927-1936 |  |  |  |  |  |  | 1991-1996 |  | 2001-2006 | 2006-2011 |
| 1932-1941 |  |  |  |  |  | 1991-1996 |  | 2001-2006 | 2006-2011 |  |
| 1937-1946 |  |  |  |  | 1991-1996 |  | 2001-2006 | 2006-2011 |  |  |
| 1942-1951 |  |  |  | 1991-1996 |  | 2001-2006 | 2006-2011 |  |  |  |
| 1947-1956 |  |  | 1991-1996 |  | 2001-2006 | 2006-2011 |  |  |  |  |
| 1952-1961 |  | 1991-1996 |  | 2001-2006 | 2006-2011 |  |  |  |  |  |
| 1957-1966 | 1991-1996 |  | 2001-2006 | 2006-2011 |  |  |  |  |  |  |
| 1962-1971 |  | 2001-2006 | 2006-2011 |  |  |  |  |  |  |  |
| 1967-1976 | 2001-2006 | 2006-2011 |  |  |  |  |  |  |  |  |
| 1972-1981 | 2006-2011 |  |  |  |  |  |  |  |  |  |

**In the same manner, we reconstructed birth cohorts for every country. The information of birth cohorts is presented in table 4.**

Table 4. Birth cohorts reconstructed for each country.

| Country | Birth cohort constructed | | | | | |
| --- | --- | --- | --- | --- | --- | --- |
| Belgium |  |  |  |  |  | 1927-1936 |
| Denmark |  |  |  |  |  | 1926-1934 |
| Estonia |  |  |  |  |  | 1928-1936 |
| Finland | 1902-1910 | 1907-1915 | 1912-1920 | 1917-1925 | 1922-1930 | 1927-1935 |
| Hungary |  | 1909-1916 | 1914-1921 | 1919-1926 | 1924-1931 | 1929-1936 |
| Italy (Turin) |  | 1907-1916 | 1912-1921 | 1917-1926 | 1922-1931 | 1927-1936 |
| Lithuania |  |  |  |  |  |  |
| Norway | 1902-1910 | 1907-1915 | 1912-1920 | 1917-1925 | 1922-1930 | 1927-1935 |
| Spain (Barcelona) |  |  |  |  | 1923-1931 | 1928-1936 |
| Sweden |  |  |  |  | 1921-1929 | 1926-1934 |
| Switzerland |  |  |  |  | 1922-1930 | 1927-1935 |
| *Cohort code* | *3* | *4* | *5* | *6* | *7* | *8* |

Continuing.

| Country | Birth cohort constructed | | | | | Number of birth years | Number of cohorts* |
| --- | --- | --- | --- | --- | --- | --- | --- |
| Belgium | 1932-1942 | 1937-1946 | 1942-1951 | 1947-1956 | 1952-1961 | 10 | 6 |
| Denmark | 1931-1939 | 1936-1944 | 1941-1949 | 1946-1954 | 1951-1959 | 9 | 6 |
| Estonia | 1933-1941 | 1938-1946 | 1943-1951 | 1948-1956 |  | 9 | 5 |
| Finland | 1932-1940 | 1937-1945 | 1942-1950 | 1947-1955 | 1952-1960 | 9 | 11 |
| Hungary | 1934-1941 | 1939-1946 | 1944-1951 | 1949-1956 |  | 8 | 9 |
| Italy (Turin) | 1932-1941 | 1937-1946 | 1942-1951 |  |  | 10 | 8 |
| Lithuania | 1932-1940 | 1937-1945 | 1942-1950 | 1947-1955 | 1952-1960 | 9 | 5 |
| Norway | 1932-1940 | 1937-1945 | 1942-1950 |  |  | 9 | 9 |
| Spain (Barcelona) | 1933-1941 | 1938-1946 | 1943-1951 | 1948-1956 | 1953-1961 | 9 | 7 |
| Sweden | 1931-1939 | 1936-1944 | 1941-1949 | 1946-1954 |  | 9 | 6 |
| Switzerland | 1932-1940 | 1937-1945 | 1942-1950 | 1947-1955 | 1952-1960 | 10 | 7 |
| *Cohort code* | *9* | *10* | *11* | *12* | *13* |  |  |

## Furthermore, we summarised in the information of age composition of each birth cohort. See file 2_b.

## Supplementary file 3. Calculations of smoking-attributable mortality from the Preston-Glei-Wilmoth method

The Preston-Glei-Wilmoth (PGW) method is a novel method developed by Preston *et al^[[15]](#footnote-15)^*. It uses lung cancer deaths as an indicator to predict deaths from other causes of death attributable to smoking, offering an estimation of the full damage from smoking.

The PGW method has a few advantages compared to other commonly used methods to assess the contribution of smoking to mortality; such as PAF and the Peto-Lopez method. Firstly, it does not rely on the information of smoking behaviors, compared to the population attributable fraction (PAF) method. The PAF method uses smoking prevalence and relative risk between smokers and non-smokers, and it has limitations in reliability of historical smoking prevalence^[[16]](#footnote-16)^ and no proxy in smoking intensity^[[17]](#footnote-17)^. Secondly, the PGW method does not rely on relative risks between smoker and non-smokers on mortality, which are used in the Peto-Lopez method and may not be generalizable across countries^[[18]](#footnote-18)^ and level of education^[[19]](#footnote-19)^. The PGW method measures statistical associations between lung cancer and other causes of death across countries and over time.

We included persons of age 30 to 79, and for those below the age of 50, we adopted the extended version of the model by Martikainen *et al.^[[20]](#footnote-20)^*.

There are three steps in the PGW method. Firstly, the fraction of lung cancer deaths attributable to smoking is calculated. Secondly, the fraction of other causes of death attributable to smoking is calculated, using parameters from a negative binomial regression model that Preston *et al.* conducted. Finally, a weighted overall attributable fraction for death from all causes is calculated. Smoking-attributable number of deaths is the result of this fraction multiplying by the all-cause number of deaths.

**Step 1.** Estimating the attributable fraction of lung cancer mortality due to smoking ($A_{L}$) by country, sex, education level, age, cohort:

$$A_{L}=\frac{M_{L}-\lambda_{L}^{N}}{M_{L}}$$

where $M_{L}$is the observed death rates from lung cancer (from observations in our data);

$\lambda_{L}^{N}$ is the expected death rate among non-smokers (from CPS-II study);

if $M_{L}-\lambda_{L}^{N}$ is negative, $A_{L}$ is set to 0.

**Step 2.** Estimating fraction of other causes of death due to smoking ($A_{o}$) by country, sex, education level, age, and cohort:

Firstly, sex- and age group-specific mortality from causes other than lung cancer ($M_{o}$) is estimated, by a log-linear function of lung cancer mortality ($M_{L}$) and other covariates:

$$\ln M_{o}=\beta_{a}X_{a}+\beta_{t}X_{t}+\beta_{c}X_{c}+\beta_{ct}\left( t\times X_{c} \right)+\beta_{L}M_{L}+\beta_{Lt}\left( t\times M_{L} \right)+\beta_{La}(M_{L}X_{a})$$

where $M_{o}$ is the death rate from causes other than lung cancer by age, sex, year of death, and population;

$X_{a}$ is a set of dummy variables for each age group;

$X_{t}$ is a set of dummy variables for each calendar year;

$X_{c}$ is a set of dummy variables for each population;

$(t\times X_{c})$ is a set of interactions between calendar year and each population dummy;

$\left( t\times M_{L} \right)$ is a set of interactions between calendar year and $M_{L}$;

$\left( M_{L}X_{a} \right)$ is a set of interactions between $M_{L}$ and each age group dummy.

This step is performed by original Preston *et al*. (for age 50+) and extended by van Raalte *et al.* (for age 50-), and does not need to be repeated. The coefficients of lung cancer mortality and related interactions terms from their studies can be adopted and adjusted to different age groups and calendar years. We borrowed their coefficients and calculated the coefficients we need ($\beta_{L}^{'}$) using age groups and calendar years from our study.

Then we estimate the attributable fraction of causes of death other than lung cancer due to smoking ($A_{o}$) by country, sex, education level, age, and period:

$$A_{o}=1-e^{-\beta_{L}^{'}(M_{L}-\lambda_{L}^{N})}$$

**Step 3.** Estimating the overall attributable number of death due to smoking (N) by country, sex, education level, age, and period:

$$N=AD=A_{L}D_{L}+A_{O}D_{O}$$

where $A$ is the overall attributable fraction of deaths due to smoking;

$D$ is the observed number of deaths from all causes combined.

## Supplementary file 4. Calculations of CCMFs

We followed *Gardner et al^[[21]](#footnote-21)^1*. with the following steps of calculations for each gender:

Firstly, we summed the person-years in all countries by sex and age as the standard population in each sex-age group. We summed the observed number of smoking-attributable deaths in all countries by sex and age as the standard number of smoking-attributable deaths in each sex-age group ($\sum D$). Secondly, we calculated the expected number of smoking-attributable deaths for each birth cohort with the standard population and observed smoking-attributable deaths rates in each country-sex-education level group ($\sum F$). Finally, we calculate the ratio of expected number smoking-attributable deaths $\sum F$ in each country-sex-education level group, to the standard number of smoking-attributable deaths $\sum D$, as the CCMF for every birth cohort in each country-sex-education level group ($CCMF=\sum F/\sum D$). We also calculated CCMFs for total mortality for later use in the same manner, using total number of deaths instead of smoking-attributable deaths. Details of the calculations can be found in the supplement file 6. The assumption behind the CCMF method is that, if we had data of any age composition or full age range (30 to 79 years of age) for each birth cohort, we would have the same CCMF results as we do here.

Each step is as followed:

**First use the following notations:**

$i=$ number of age groups (table 1-2, rows);

$j=$ number of calendar periods of time (table 1-2, columns);

$e=$ level of education;

$c=$ number of countries;

$k=$ number of cohorts;

$d_{ijec}=$ number of deaths in age group $i$ during period $j$, level of education $e$, and country $c$;

$y_{ijec}=$ person-years in age group $i$ during period $j$, level of education $e$, and country $c$;

$r_{ijec}=\frac{d_{ijec}}{y_{ijec}}=$ smoking-attributable death rate in age group $i$ during period $j$, level of education $e$, and country $c$.

**Secondly, we define:**

Population structure in standard population ($Y_{i}$): person-years summed across in all 11 countries by age;

Age-specific death rates in standard population ($R_{i}$): number of smoking-attributable deaths summed in all 11 countries by age ($D_{i}$) divided by age-specific standard population ($R_{i}=D_{i}/Y_{i}$).

**Then, we define:**

$$f_{ijec}=Y_{i}r_{ijec}$$

to be the expected number of deaths if persons in age-group $i$ of the standard population would have been exposed to the observed death rate in age group $i$ during cohort $k$, level of education $e$, and country $c$;

$$F_{kec}=\sum_{i} f_{ikec}$$

To be the expected number of deaths for a specific birth cohort $k$ in level of education $e$, and country $c$;

and

$$D_{k}=\sum_{i} D_{i}$$

to be the expected number of deaths for a specific birth cohort $k$ if they had standard population structure and death rates in the same age group.

**Finally, the comparative cohort mortality figure (CCMF) for each birth cohort is given by:**

$$\frac{Expected deaths in standard population at death rates of cohort k, education level e, and country c}{Expected deaths in standard at standard rates}$$

=

$$\frac{F_{kec}}{D_{k}}$$

so that

$$CCMF=\frac{F_{kec}}{D_{k}}=\sum_{i} \frac{D_{i}}{D_{k}}\frac{r_{ijec}}{R_{i}}$$

as CCMF is the weighted arithmetic means of age-specific rate ratios.

Supplementary file 5. CCMF results from less strict exclusion criteria.

When we exclude number of lung cancer deaths is less than 10 by country, sex, education level, age, and cohort; and birth cohorts with data less than two age groups (instead of three age groups), we got the following results.


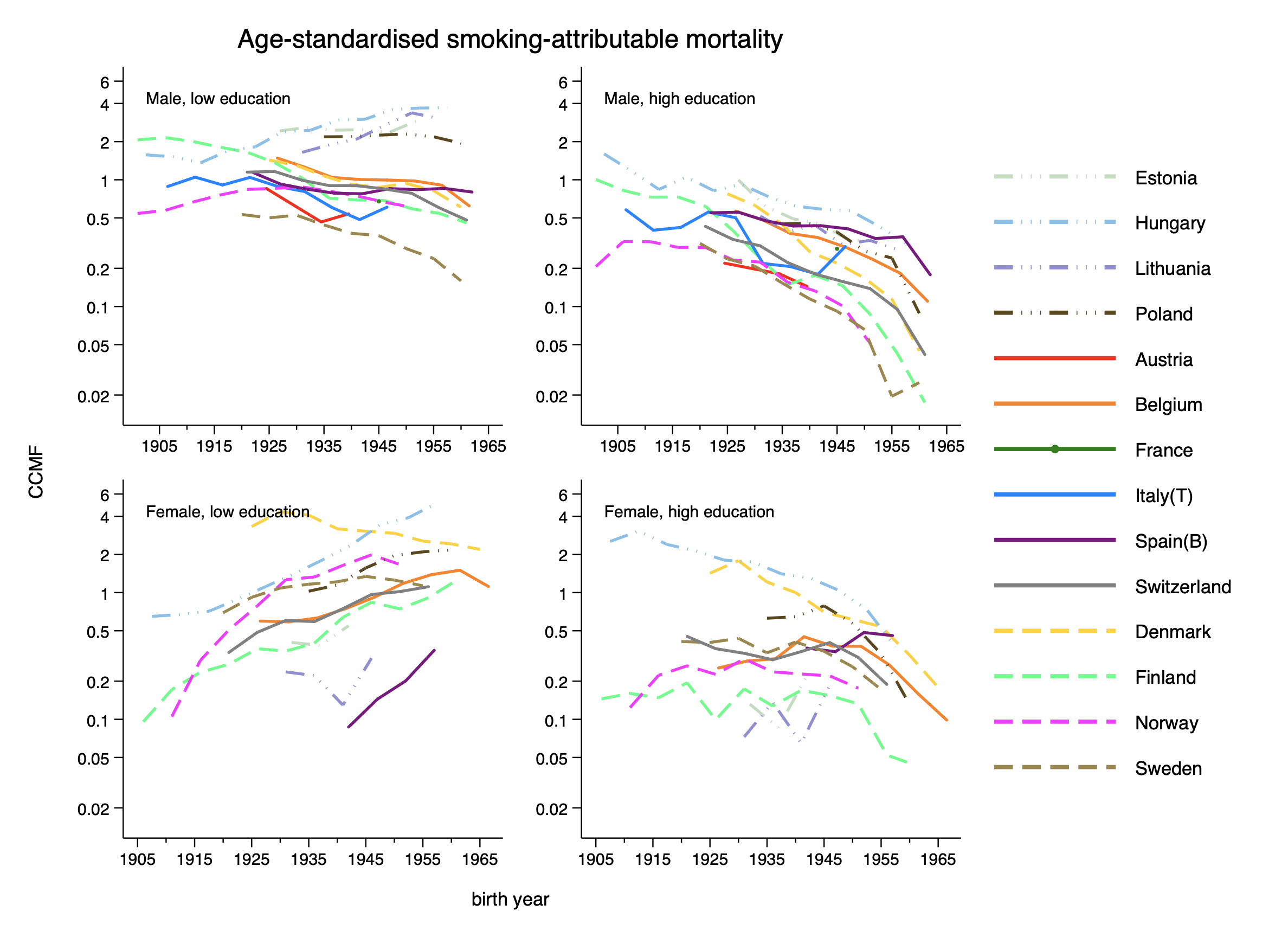


Figure 1. Comparative cohort mortality figure of smoking-attributable mortality plotted vs the (median) year of birth.


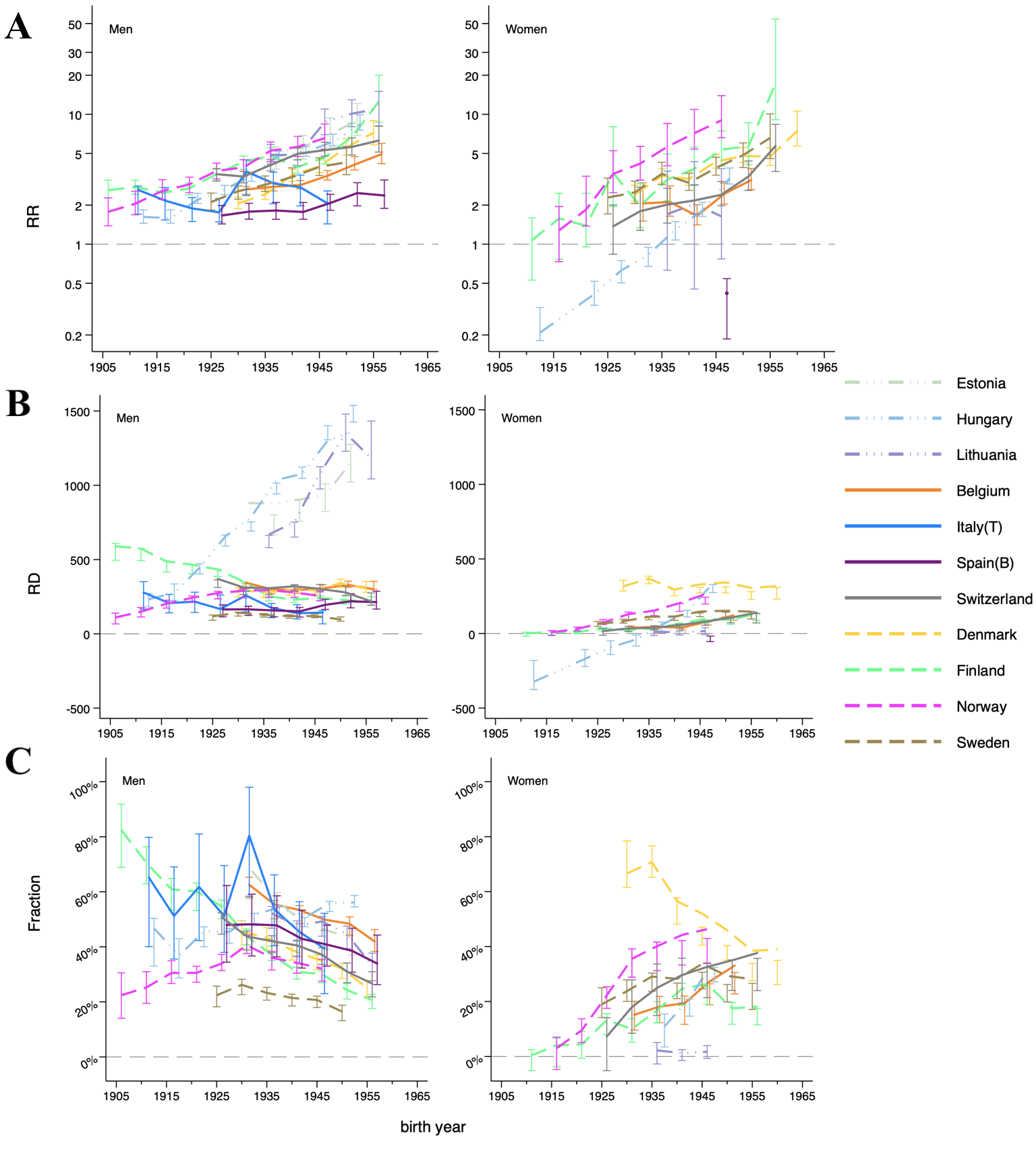


Figure 2. Relative inequalities (A), absolute inequalities (B) in smoking-attributable mortality, contribution of smoking to absolute inequalities in total mortality (C)*, in 11 European countries by sex and by educational level.

*In Figure 2-C, we had excluded generations born before 1902 in Finland, before 1929 in Hungary, before 1907 in Norway, before 1953 in Spain and before 1917 in Switzerland. Because they had higher SAM among the high-educated and higher total mortality rates among the low-educated in older generation

After applying for less strict exclusion criteria, we were able to present some results on Austria and on Poland. We were also able to present more recent birth cohort, mostly one or two generations younger than in our main results. But the overall results remained largely the same as in the main results.

# Supplementary file 6. Birth cohort-specific trends of total mortality

We would like to know the reason for the decreasing contribution of inequalities in SAM to inequalities in total mortality among men in consecutive generations, while absolute inequalities remained stable. We plotted CCMF for total mortality, following the same procedure in the CCMF method (Figure 3-4).


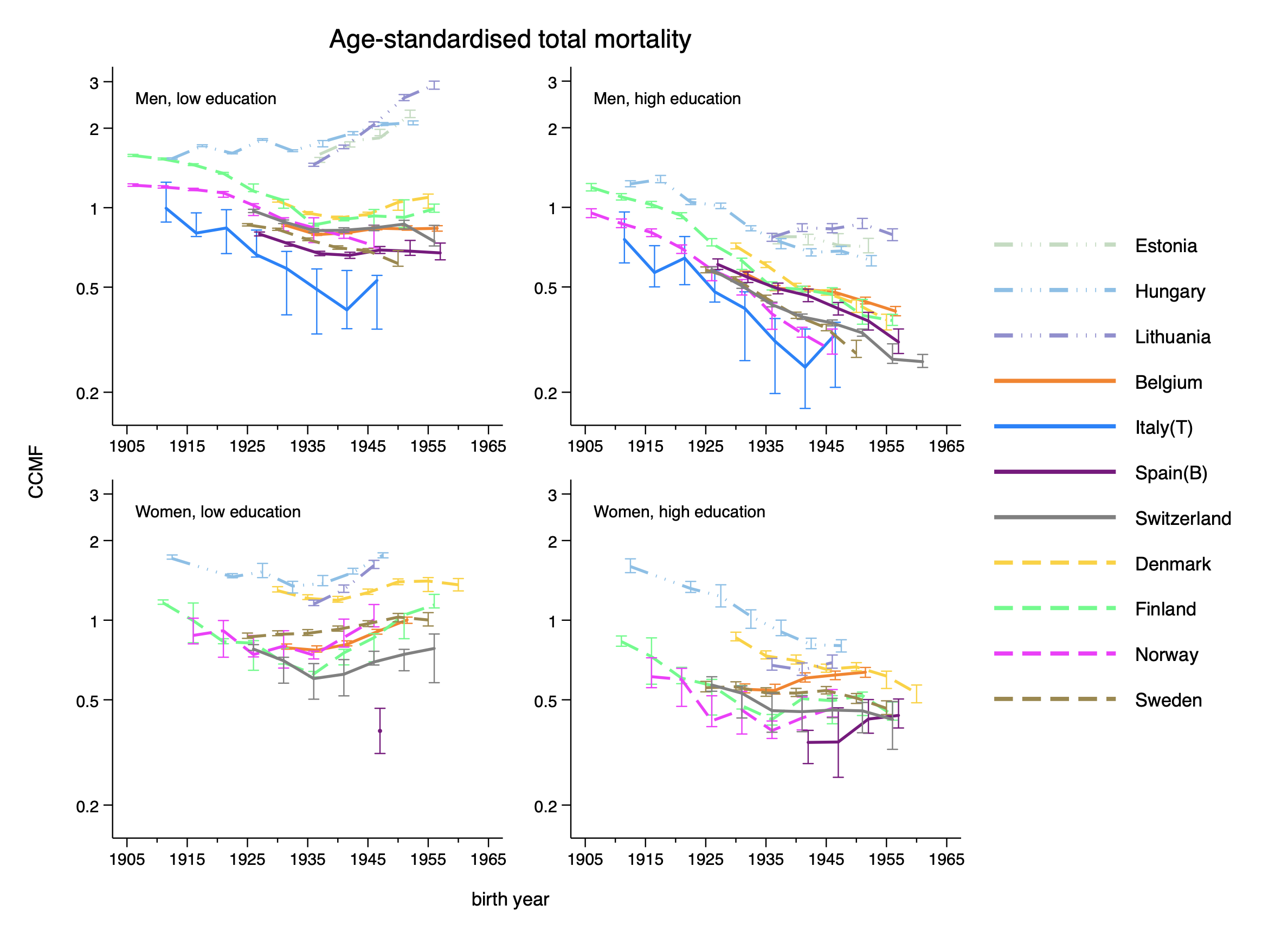


Figure 3. Comparative cohort mortality figure of total mortality plotted vs the (median) year of birth.


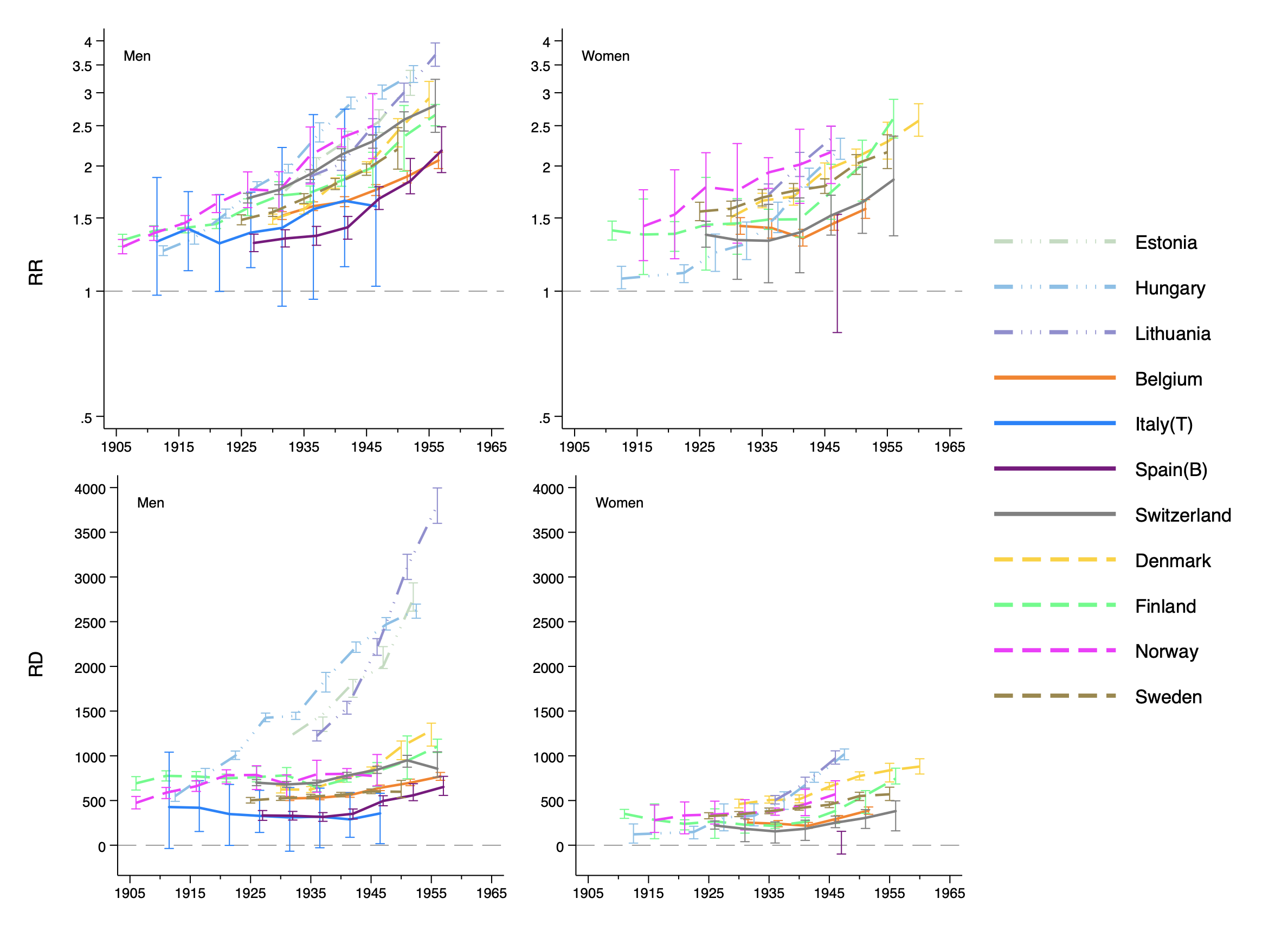


Figure 4. Rate ratios and rate difference (per 100,000 person-years) between the low- and the high-educated.

Among the low-educated men and women, we saw U-shaped trends in consecutive birth cohorts in most countries, where generations born around 1941 reached the lowest total mortality rates. Among the high-educated men and women, total mortality decreased in consecutive birth cohorts in most countries. In terms of absolute inequalities in total mortality, we saw increases among both men and women in consecutive birth cohorts.

Even though we might conclude that smoking will become less important as a driver of inequalities in mortality in the future, the increasing absolute inequalities in total mortality would still be a worrying problem that joint efforts should be devoted to.

# Supplementary file 7. Table for rate ratio, rate difference and the fraction with 95% CI.

Table 6. exact number of rate ratio, rate difference, and fraction of inequality in smoking-attributable mortality to inequality in total mortality, with 95% CI, in each country

|  |  |  |  | 95% CI | |  | 95% CI | |  | 95% CI | |
| --- | --- | --- | --- | --- | --- | --- | --- | --- | --- | --- | --- |
| Country | sex | cohort | RR | upper | lower | RD | upper | lower | fraction | upper | lower |
| Belgium | Males | 1927-1935 | 2.6 | 2.4 | 2.8 | 320.0 | 302.7 | 335.9 | 0.61 | 0.58 | 0.65 |
| Belgium | Males | 1932-1940 | 2.8 | 2.6 | 3.0 | 283.1 | 268.0 | 298.2 | 0.53 | 0.50 | 0.57 |
| Belgium | Males | 1937-1945 | 2.9 | 2.7 | 3.1 | 289.2 | 272.6 | 305.7 | 0.52 | 0.48 | 0.55 |
| Belgium | Males | 1942-1950 | 3.4 | 3.1 | 3.7 | 313.8 | 296.4 | 330.9 | 0.49 | 0.46 | 0.52 |
| Belgium | Males | 1947-1955 | 4.2 | 3.7 | 4.7 | 331.3 | 312.4 | 353.2 | 0.47 | 0.44 | 0.51 |
| Belgium | Males | 1952-1960 | 5.0 | 4.2 | 5.9 | 327.4 | 297.3 | 353.6 | 0.43 | 0.39 | 0.46 |
| Belgium | Females | 1927-1935 | 2.0 | 1.5 | 3.0 | 38.3 | 25.0 | 50.0 | 0.15 | 0.10 | 0.20 |
| Belgium | Females | 1932-1940 | 2.1 | 1.6 | 2.8 | 41.7 | 29.7 | 52.9 | 0.17 | 0.12 | 0.23 |
| Belgium | Females | 1937-1945 | 1.7 | 1.4 | 2.0 | 37.4 | 25.6 | 48.8 | 0.17 | 0.12 | 0.23 |
| Belgium | Females | 1942-1950 | 2.4 | 2.0 | 2.9 | 67.6 | 56.7 | 78.4 | 0.22 | 0.19 | 0.26 |
| Belgium | Females | 1947-1955 | 3.1 | 2.6 | 3.8 | 101.8 | 88.5 | 116.0 | 0.26 | 0.22 | 0.31 |
| Denmark | Males | 1927-1935 | 2.1 | 1.9 | 2.3 | 279.3 | 248.7 | 310.5 | 0.45 | 0.40 | 0.50 |
| Denmark | Males | 1932-1940 | 2.4 | 2.2 | 2.6 | 264.4 | 242.2 | 289.0 | 0.42 | 0.39 | 0.47 |
| Denmark | Males | 1937-1945 | 3.4 | 3.1 | 3.8 | 278.1 | 260.7 | 298.2 | 0.38 | 0.35 | 0.41 |
| Denmark | Males | 1942-1950 | 4.0 | 3.6 | 4.4 | 287.0 | 267.6 | 308.5 | 0.34 | 0.32 | 0.37 |
| Denmark | Males | 1947-1955 | 5.6 | 4.9 | 6.5 | 341.5 | 314.8 | 370.3 | 0.31 | 0.28 | 0.35 |
| Denmark | Males | 1952-1960 | 7.1 | 5.8 | 9.3 | 316.6 | 279.1 | 354.5 | 0.25 | 0.22 | 0.28 |
| Denmark | Females | 1927-1935 | 2.5 | 2.2 | 2.9 | 331.4 | 285.0 | 360.3 | 0.72 | 0.60 | 0.81 |
| Denmark | Females | 1932-1940 | 3.4 | 2.9 | 3.8 | 368.6 | 334.2 | 386.2 | 0.73 | 0.64 | 0.78 |
| Denmark | Females | 1937-1945 | 3.2 | 2.8 | 3.6 | 277.4 | 252.9 | 293.1 | 0.54 | 0.47 | 0.59 |
| Denmark | Females | 1942-1950 | 4.3 | 3.8 | 4.9 | 293.6 | 270.9 | 309.5 | 0.45 | 0.40 | 0.48 |
| Denmark | Females | 1947-1955 | 4.8 | 4.2 | 5.5 | 293.1 | 272.2 | 313.4 | 0.38 | 0.35 | 0.41 |
| Denmark | Females | 1952-1960 | 4.7 | 4.0 | 5.5 | 254.5 | 225.6 | 281.9 | 0.30 | 0.27 | 0.35 |
| Denmark | Females | 1957-1965 | 7.7 | 5.8 | 10.3 | 270.8 | 231.6 | 305.3 | 0.31 | 0.27 | 0.35 |
| Estonia | Males | 1932-1940 | 5.0 | 3.8 | 5.6 | 814.4 | 669.8 | 806.6 | 0.56 | 0.49 | 0.60 |
| Estonia | Males | 1937-1945 | 5.5 | 4.5 | 6.8 | 872.7 | 760.7 | 900.7 | 0.48 | 0.44 | 0.52 |
| Estonia | Males | 1942-1950 | 7.0 | 5.6 | 9.2 | 895.0 | 837.7 | 1002.5 | 0.45 | 0.40 | 0.48 |
| Estonia | Males | 1947-1955 | 9.0 | 6.9 | 12.0 | 1150.2 | 1029.7 | 1283.2 | 0.42 | 0.37 | 0.46 |
| Finland | Males | 1902-1910 | 2.6 | 2.3 | 3.1 | 551.5 | 495.3 | 608.5 | 0.80 | 0.69 | 0.92 |
| Finland | Males | 1907-1915 | 2.8 | 2.5 | 3.1 | 535.9 | 496.7 | 577.7 | 0.69 | 0.63 | 0.76 |
| Finland | Males | 1912-1920 | 2.5 | 2.2 | 2.8 | 452.0 | 416.4 | 490.8 | 0.59 | 0.53 | 0.65 |
| Finland | Males | 1917-1925 | 2.6 | 2.4 | 3.0 | 434.2 | 403.5 | 466.4 | 0.58 | 0.54 | 0.63 |
| Finland | Males | 1922-1930 | 3.4 | 3.0 | 3.8 | 407.2 | 384.9 | 436.1 | 0.53 | 0.49 | 0.58 |
| Finland | Males | 1927-1935 | 4.3 | 3.7 | 4.9 | 337.1 | 307.3 | 355.2 | 0.43 | 0.39 | 0.50 |
| Finland | Males | 1932-1940 | 4.8 | 4.1 | 5.5 | 240.1 | 221.0 | 254.7 | 0.37 | 0.34 | 0.41 |
| Finland | Males | 1937-1945 | 3.9 | 3.5 | 4.5 | 224.5 | 208.8 | 239.7 | 0.30 | 0.28 | 0.33 |
| Finland | Males | 1942-1950 | 4.7 | 4.1 | 5.5 | 239.3 | 220.3 | 257.8 | 0.29 | 0.26 | 0.33 |
| Finland | Males | 1947-1955 | 6.8 | 5.6 | 8.6 | 220.9 | 199.6 | 254.6 | 0.23 | 0.20 | 0.29 |
| Finland | Males | 1952-1960 | 12.8 | 8.8 | 19.7 | 222.0 | 193.8 | 252.4 | 0.20 | 0.17 | 0.23 |
| Finland | Females | 1907-1915 | 1.1 | 0.6 | 1.6 | 1.6 | -15.5 | 9.5 | 0.00 | -0.05 | 0.03 |
| Finland | Females | 1912-1920 | 1.6 | 0.7 | 2.7 | 11.8 | -11.1 | 20.7 | 0.04 | -0.08 | 0.08 |
| Finland | Females | 1917-1925 | 1.4 | 0.9 | 2.5 | 10.8 | -2.9 | 23.3 | 0.05 | -0.01 | 0.09 |
| Finland | Females | 1922-1930 | 3.7 | 1.7 | 10.2 | 34.7 | 15.1 | 42.8 | 0.13 | 0.08 | 0.27 |
| Finland | Females | 1927-1935 | 2.0 | 1.3 | 3.2 | 23.1 | 9.9 | 33.8 | 0.10 | 0.05 | 0.15 |
| Finland | Females | 1932-1940 | 3.1 | 2.1 | 4.9 | 35.8 | 26.1 | 42.8 | 0.17 | 0.12 | 0.20 |
| Finland | Females | 1937-1945 | 3.8 | 2.8 | 5.5 | 59.9 | 48.2 | 68.1 | 0.23 | 0.18 | 0.29 |
| Finland | Females | 1942-1950 | 5.5 | 3.9 | 7.6 | 85.1 | 69.0 | 93.0 | 0.22 | 0.17 | 0.30 |
| Finland | Females | 1947-1955 | 5.6 | 4.1 | 8.7 | 76.3 | 62.1 | 88.7 | 0.14 | 0.12 | 0.20 |
| Finland | Females | 1952-1960 | 17.5 | 8.9 | 58.6 | 108.0 | 87.1 | 132.4 | 0.14 | 0.11 | 0.18 |
| Hungary | Males | 1907-1915 | 1.6 | 1.4 | 1.8 | 225.9 | 182.2 | 271.7 | 0.41 | 0.33 | 0.50 |
| Hungary | Males | 1912-1920 | 1.6 | 1.5 | 1.8 | 279.8 | 226.3 | 333.8 | 0.36 | 0.29 | 0.44 |
| Hungary | Males | 1917-1925 | 2.2 | 2.1 | 2.5 | 435.5 | 404.7 | 474.6 | 0.43 | 0.40 | 0.47 |
| Hungary | Males | 1922-1930 | 2.6 | 2.4 | 2.8 | 626.7 | 591.3 | 667.5 | 0.44 | 0.41 | 0.47 |
| Hungary | Males | 1927-1935 | 3.3 | 3.0 | 3.5 | 721.8 | 693.0 | 754.5 | 0.50 | 0.48 | 0.52 |
| Hungary | Males | 1932-1940 | 4.7 | 4.3 | 5.2 | 980.1 | 936.3 | 1014.9 | 0.53 | 0.51 | 0.56 |
| Hungary | Males | 1937-1945 | 5.2 | 4.7 | 5.6 | 1084.4 | 1048.5 | 1124.3 | 0.49 | 0.47 | 0.51 |
| Hungary | Males | 1942-1950 | 6.3 | 5.7 | 7.1 | 1349.6 | 1305.1 | 1402.6 | 0.55 | 0.53 | 0.57 |
| Hungary | Males | 1947-1955 | 8.7 | 7.5 | 9.9 | 1486.0 | 1422.7 | 1540.8 | 0.56 | 0.55 | 0.59 |
| Hungary | Females | 1907-1915 | 0.2 | 0.2 | 0.3 | -285.5 | -378.5 | -181.0 | -2.34 | -11.04 | -0.93 |
| Hungary | Females | 1917-1925 | 0.4 | 0.3 | 0.5 | -161.4 | -220.7 | -108.2 | -1.09 | -2.66 | -0.61 |
| Hungary | Females | 1922-1930 | 0.6 | 0.5 | 0.7 | -87.6 | -143.0 | -46.9 | -0.28 | -0.71 | -0.13 |
| Hungary | Females | 1927-1935 | 0.8 | 0.7 | 1.0 | -49.1 | -86.5 | -7.0 | -0.15 | -0.32 | -0.02 |
| Hungary | Females | 1932-1940 | 1.3 | 1.1 | 1.5 | 50.8 | 15.9 | 80.0 | 0.10 | 0.03 | 0.16 |
| Hungary | Females | 1937-1945 | 1.8 | 1.6 | 2.1 | 134.9 | 108.8 | 157.9 | 0.18 | 0.15 | 0.21 |
| Hungary | Females | 1942-1950 | 3.3 | 2.9 | 3.8 | 302.8 | 274.5 | 329.8 | 0.30 | 0.27 | 0.32 |
| Italy(T) | Males | 1907-1915 | 2.6 | 1.5 | 3.3 | 280.5 | 132.2 | 405.1 | 0.66 | -2.22 | 4.03 |
| Italy(T) | Males | 1912-1920 | 2.2 | 1.4 | 2.8 | 214.6 | 116.3 | 297.7 | 0.51 | 0.33 | 0.93 |
| Italy(T) | Males | 1917-1925 | 1.9 | 1.3 | 2.6 | 210.9 | 93.2 | 328.2 | 0.61 | 0.04 | 1.50 |
| Italy(T) | Males | 1922-1930 | 1.8 | 1.4 | 2.5 | 168.2 | 110.9 | 282.8 | 0.51 | 0.38 | 0.87 |
| Italy(T) | Males | 1927-1935 | 3.7 | 1.8 | 5.9 | 255.8 | 110.7 | 330.5 | 0.82 | -3.71 | 4.69 |
| Italy(T) | Males | 1932-1940 | 2.9 | 1.5 | 5.0 | 170.7 | 66.9 | 252.3 | 0.53 | -1.68 | 2.52 |
| Italy(T) | Males | 1937-1945 | 2.7 | 1.6 | 4.3 | 130.4 | 74.5 | 231.9 | 0.45 | 0.32 | 1.02 |
| Italy(T) | Males | 1942-1950 | 2.0 | 1.3 | 3.1 | 135.6 | 37.4 | 184.9 | 0.38 | 0.20 | 1.10 |
| Lithuania | Males | 1932-1940 | 4.9 | 4.1 | 5.8 | 620.4 | 579.0 | 664.4 | 0.51 | 0.47 | 0.55 |
| Lithuania | Males | 1937-1945 | 4.7 | 4.0 | 5.7 | 700.1 | 645.4 | 749.8 | 0.46 | 0.42 | 0.49 |
| Lithuania | Males | 1942-1950 | 8.7 | 7.1 | 11.0 | 1043.1 | 979.5 | 1120.4 | 0.47 | 0.44 | 0.51 |
| Lithuania | Males | 1947-1955 | 10.1 | 8.1 | 12.9 | 1343.3 | 1225.6 | 1471.8 | 0.43 | 0.39 | 0.48 |
| Lithuania | Males | 1952-1960 | 10.8 | 8.2 | 15.3 | 1237.4 | 1047.4 | 1438.3 | 0.33 | 0.28 | 0.38 |
| Lithuania | Females | 1932-1940 | 1.5 | 0.7 | 3.7 | 9.8 | -11.2 | 24.4 | 0.02 | -0.02 | 0.05 |
| Lithuania | Females | 1937-1945 | 1.9 | 0.5 | 5.3 | 7.9 | -10.6 | 19.2 | 0.01 | -0.01 | 0.03 |
| Lithuania | Females | 1942-1950 | 1.6 | 0.7 | 3.4 | 14.9 | -8.8 | 38.4 | 0.02 | -0.01 | 0.04 |
| Norway | Males | 1902-1910 | 1.7 | 1.4 | 2.3 | 102.4 | 67.6 | 137.2 | 0.22 | 0.14 | 0.30 |
| Norway | Males | 1907-1915 | 2.1 | 1.7 | 2.6 | 146.0 | 114.0 | 175.9 | 0.25 | 0.19 | 0.31 |
| Norway | Males | 1912-1920 | 2.6 | 2.2 | 3.2 | 201.2 | 173.0 | 227.6 | 0.30 | 0.26 | 0.36 |
| Norway | Males | 1917-1925 | 2.9 | 2.5 | 3.3 | 235.3 | 211.6 | 257.6 | 0.30 | 0.27 | 0.34 |
| Norway | Males | 1922-1930 | 3.7 | 3.1 | 4.3 | 267.9 | 234.0 | 287.6 | 0.34 | 0.30 | 0.41 |
| Norway | Males | 1927-1935 | 3.9 | 3.3 | 4.6 | 280.5 | 238.5 | 303.0 | 0.41 | 0.36 | 0.48 |
| Norway | Males | 1932-1940 | 5.2 | 4.2 | 6.3 | 281.9 | 238.3 | 308.8 | 0.36 | 0.31 | 0.42 |
| Norway | Males | 1937-1945 | 5.6 | 4.7 | 6.8 | 267.9 | 247.5 | 293.2 | 0.34 | 0.30 | 0.37 |
| Norway | Males | 1942-1950 | 6.6 | 5.2 | 8.5 | 246.9 | 221.9 | 291.6 | 0.32 | 0.26 | 0.38 |
| Norway | Females | 1912-1920 | 1.4 | 0.7 | 2.0 | 10.6 | -15.5 | 19.8 | 0.04 | -0.10 | 0.07 |
| Norway | Females | 1917-1925 | 2.0 | 1.2 | 3.8 | 33.7 | 9.5 | 49.1 | 0.10 | 0.04 | 0.16 |
| Norway | Females | 1922-1930 | 3.5 | 2.3 | 5.6 | 75.9 | 58.0 | 99.7 | 0.22 | 0.17 | 0.29 |
| Norway | Females | 1927-1935 | 4.3 | 2.7 | 6.2 | 128.0 | 82.6 | 151.6 | 0.35 | 0.26 | 0.51 |
| Norway | Females | 1932-1940 | 5.6 | 4.0 | 8.4 | 139.3 | 118.5 | 154.5 | 0.37 | 0.31 | 0.43 |
| Norway | Females | 1937-1945 | 7.2 | 5.2 | 11.0 | 175.8 | 147.9 | 202.3 | 0.38 | 0.31 | 0.49 |
| Norway | Females | 1942-1950 | 9.0 | 6.6 | 13.7 | 219.2 | 197.3 | 244.8 | 0.38 | 0.32 | 0.46 |
| Spain(B) | Males | 1922-1930 | 1.7 | 1.5 | 2.0 | 155.6 | 117.8 | 195.2 | 0.47 | 0.35 | 0.61 |
| Spain(B) | Males | 1927-1935 | 1.8 | 1.6 | 2.0 | 153.8 | 124.5 | 184.6 | 0.47 | 0.38 | 0.58 |
| Spain(B) | Males | 1932-1940 | 1.8 | 1.6 | 2.1 | 148.1 | 116.6 | 178.9 | 0.47 | 0.37 | 0.59 |
| Spain(B) | Males | 1937-1945 | 1.8 | 1.5 | 2.1 | 146.7 | 111.9 | 179.6 | 0.42 | 0.32 | 0.53 |
| Spain(B) | Males | 1942-1950 | 2.1 | 1.8 | 2.4 | 196.9 | 165.2 | 228.6 | 0.40 | 0.34 | 0.48 |
| Spain(B) | Males | 1947-1955 | 2.4 | 2.0 | 3.0 | 217.3 | 174.6 | 267.4 | 0.39 | 0.31 | 0.47 |
| Spain(B) | Males | 1952-1960 | 2.4 | 1.9 | 3.2 | 226.2 | 165.5 | 288.3 | 0.35 | 0.26 | 0.44 |
| Spain(B) | Females | 1942-1950 | 0.4 | 0.2 | 0.6 | -24.5 | -54.3 | -16.8 | -0.66 | -4.22 | 7.93 |
| Sweden | Males | 1922-1930 | 2.1 | 1.8 | 2.5 | 109.4 | 93.4 | 126.6 | 0.22 | 0.18 | 0.25 |
| Sweden | Males | 1927-1935 | 2.5 | 2.2 | 2.8 | 132.8 | 119.2 | 145.9 | 0.25 | 0.23 | 0.28 |
| Sweden | Males | 1932-1940 | 2.9 | 2.5 | 3.3 | 123.6 | 112.7 | 135.7 | 0.23 | 0.21 | 0.25 |
| Sweden | Males | 1937-1945 | 3.3 | 2.9 | 3.9 | 116.0 | 104.7 | 128.8 | 0.21 | 0.18 | 0.23 |
| Sweden | Males | 1942-1950 | 4.0 | 3.4 | 4.7 | 120.2 | 108.7 | 133.9 | 0.20 | 0.18 | 0.22 |
| Sweden | Males | 1947-1955 | 4.4 | 3.4 | 5.7 | 97.8 | 83.9 | 114.8 | 0.16 | 0.13 | 0.19 |
| Sweden | Females | 1922-1930 | 2.3 | 1.7 | 3.3 | 66.1 | 47.6 | 82.1 | 0.20 | 0.14 | 0.25 |
| Sweden | Females | 1927-1935 | 2.5 | 2.1 | 3.0 | 84.7 | 70.1 | 96.6 | 0.25 | 0.20 | 0.29 |
| Sweden | Females | 1932-1940 | 3.5 | 2.9 | 4.3 | 105.3 | 91.6 | 115.4 | 0.28 | 0.23 | 0.31 |
| Sweden | Females | 1937-1945 | 3.0 | 2.6 | 3.5 | 101.3 | 88.2 | 111.2 | 0.24 | 0.21 | 0.27 |
| Sweden | Females | 1942-1950 | 3.9 | 3.4 | 4.6 | 125.2 | 111.7 | 137.3 | 0.28 | 0.24 | 0.31 |
| Sweden | Females | 1947-1955 | 4.8 | 3.9 | 6.0 | 125.7 | 109.1 | 143.2 | 0.23 | 0.20 | 0.26 |
| Sweden | Females | 1952-1960 | 6.6 | 4.7 | 10.4 | 122.3 | 97.4 | 149.1 | 0.21 | 0.17 | 0.26 |
| Switzerland | Males | 1922-1930 | 3.4 | 3.1 | 3.8 | 339.1 | 314.7 | 363.1 | 0.48 | 0.45 | 0.52 |
| Switzerland | Males | 1927-1935 | 3.3 | 3.0 | 3.6 | 287.3 | 266.9 | 308.5 | 0.42 | 0.39 | 0.46 |
| Switzerland | Males | 1932-1940 | 4.0 | 3.7 | 4.5 | 283.6 | 266.0 | 304.3 | 0.41 | 0.38 | 0.44 |
| Switzerland | Males | 1937-1945 | 5.0 | 4.5 | 5.5 | 307.3 | 285.8 | 329.9 | 0.39 | 0.36 | 0.42 |
| Switzerland | Males | 1942-1950 | 5.4 | 4.8 | 6.1 | 307.0 | 281.9 | 334.2 | 0.36 | 0.34 | 0.40 |
| Switzerland | Males | 1947-1955 | 5.6 | 4.8 | 6.6 | 289.2 | 257.9 | 321.4 | 0.30 | 0.27 | 0.34 |
| Switzerland | Males | 1952-1960 | 6.4 | 5.1 | 8.2 | 226.8 | 193.5 | 276.5 | 0.26 | 0.22 | 0.32 |
| Switzerland | Females | 1922-1930 | 1.3 | 0.8 | 2.0 | 14.7 | -10.6 | 30.7 | 0.07 | -0.05 | 0.14 |
| Switzerland | Females | 1927-1935 | 1.8 | 1.1 | 3.4 | 35.7 | 3.5 | 54.3 | 0.19 | 0.04 | 0.45 |
| Switzerland | Females | 1932-1940 | 2.0 | 1.3 | 3.1 | 37.9 | 14.6 | 60.1 | 0.24 | 0.13 | 0.51 |
| Switzerland | Females | 1937-1945 | 2.2 | 1.6 | 3.1 | 51.4 | 29.3 | 66.1 | 0.28 | 0.19 | 0.63 |
| Switzerland | Females | 1942-1950 | 2.4 | 2.0 | 3.1 | 69.8 | 55.3 | 84.4 | 0.28 | 0.21 | 0.36 |
| Switzerland | Females | 1947-1955 | 3.3 | 2.5 | 4.5 | 89.2 | 68.5 | 104.8 | 0.29 | 0.22 | 0.42 |
| Switzerland | Females | 1952-1960 | 5.9 | 3.3 | 8.4 | 116.7 | 70.0 | 134.7 | 0.31 | 0.23 | 0.44 |

1. Department of Public Health, Erasmus MC, Rotterdam, The Netherlands

   Email: d.long@erasmusmc.nl [↑](#footnote-ref-1)
2. Department of Public Health, Erasmus MC, Rotterdam, The Netherlands [↑](#footnote-ref-2)
3. Population Research Unit, Faculty of Social Sciences, University of Helsinki, Helsinki, Finland [↑](#footnote-ref-3)
4. Department of Public Health Sciences, Stockholm University, Sweden [↑](#footnote-ref-4)
5. Department of Public Health, University of Copenhagen, Copenhagen, Denmark [↑](#footnote-ref-5)
6. Epidemiology, Biostatistics and Prevention Institute, University of Zurich, Zurich, Switzerland [↑](#footnote-ref-6)
7. Social Medicine and global Health, Lund University, Malmö, Sweden [↑](#footnote-ref-7)
8. Clinica Ortopedica e Traumatologica II, Istituto Ortopedico Rizzoli, Bologna, Italy [↑](#footnote-ref-8)
9. Demographic Research Institute, Budapest, Hungary [↑](#footnote-ref-9)
10. Stockholm Centre for Health and Social Change, Södertörn University, Huddinge, Sweden [↑](#footnote-ref-10)
11. Department of Epidemiology and Biostatistics, National Institute for Health Development, Tallinn, Estonia [↑](#footnote-ref-11)
12. Agència de Salut Pública de Barcelona, 08023 Barcelona, Spain. [↑](#footnote-ref-12)
13. CIBER Epidemiología y Salud Pública (CIBERESP), 28029 Madrid, Spain. [↑](#footnote-ref-13)
14. Department of Health Management, Faculty of Public Health, Medical Academy, Lithuanian University of Health Sciences, Lithuania [↑](#footnote-ref-14)
15. Preston, S. H., Glei, D. A., & Wilmoth, J. R. (2010). A new method for estimating smoking-attributable mortality in high-income countries. Int J Epidemiol, 39(2), 430-438. doi: dyp360 [pii]10.1093/ije/dyp360 [↑](#footnote-ref-15)
16. Nicolaides-Bouman, Ans. *International smoking statistics: a collection of historical data from 22 economically developed countries*. Oxford University Press, USA, 1993. [↑](#footnote-ref-16)
17. Ezzati, Majid, and Alan D. Lopez. "Measuring the accumulated hazards of smoking: global and regional estimates for 2000." *Tobacco Control* 12.1 (2003): 79-85. [↑](#footnote-ref-17)
18. Sterling, Theodor D., Wilf L. Rosenbaum, and James J. Weinkam. "Risk attribution and tobacco-related deaths." *American Journal of Epidemiology* 138.2 (1993): 128-139. [↑](#footnote-ref-18)
19. Wagenknecht, Lynne E., et al. "Misclassification of smoking status in the CARDIA study: a comparison of self-report with serum cotinine levels." *American Journal of Public Health* 82.1 (1992): 33-36. [↑](#footnote-ref-19)
20. Martikainen, Pekka, et al. "Income differences in life expectancy: the changing contribution of harmful consumption of alcohol and smoking." *Epidemiology* (2014): 182-190. [↑](#footnote-ref-20)
21. 1 Gardner, M. J., and C. Osmond. "Interpretation of time trends in disease rates in the presence of generation effects." *Statistics in medicine* 3.2 (1984): 113-130. [↑](#footnote-ref-21)
