# Supplementary material for: Smoking and inequalities in mortality in 11 European countries: a birth cohort analysis
Source: Popul Health Metr. 2021 Jan 30;19:3. doi: 10.1186/s12963-021-00247-2 (PMC7847590; doi:10.1186/s12963-021-00247-2)
Supplement: Supplementary file 2 — Additional file 2. [file 12963_2021_247_MOESM2_ESM.docx]

Table 5. Age composition of each birth cohort

| Country | Sex | Attained education level in 3 categories | birth cohort | min age group | max age group | number of age groups | Age category | | | | | | | | | |
| --- | --- | --- | --- | --- | --- | --- | --- | --- | --- | --- | --- | --- | --- | --- | --- | --- |
| Belgium | Males | low | 1927-1935 | 60-64 | 75-79 | 3 |  |  |  |  |  |  | 60-64 |  | 70-74 | 75-79 |
| Belgium | Males | low | 1932-1940 | 55-59 | 70-74 | 3 |  |  |  |  |  | 55-59 |  | 65-69 | 70-74 |  |
| Belgium | Males | low | 1937-1945 | 50-54 | 65-69 | 3 |  |  |  |  | 50-54 |  | 60-64 | 65-69 |  |  |
| Belgium | Males | low | 1942-1950 | 45-49 | 60-64 | 3 |  |  |  | 45-49 |  | 55-59 | 60-64 |  |  |  |
| Belgium | Males | low | 1947-1955 | 40-44 | 55-59 | 3 |  |  | 40-44 |  | 50-54 | 55-59 |  |  |  |  |
| Belgium | Males | low | 1952-1960 | 35-39 | 50-54 | 3 |  | 35-39 |  | 45-49 | 50-54 |  |  |  |  |  |
| Belgium | Males | high | 1927-1935 | 60-64 | 75-79 | 3 |  |  |  |  |  |  | 60-64 |  | 70-74 | 75-79 |
| Belgium | Males | high | 1932-1940 | 55-59 | 70-74 | 3 |  |  |  |  |  | 55-59 |  | 65-69 | 70-74 |  |
| Belgium | Males | high | 1937-1945 | 50-54 | 65-69 | 3 |  |  |  |  | 50-54 |  | 60-64 | 65-69 |  |  |
| Belgium | Males | high | 1942-1950 | 45-49 | 60-64 | 3 |  |  |  | 45-49 |  | 55-59 | 60-64 |  |  |  |
| Belgium | Males | high | 1947-1955 | 40-44 | 55-59 | 3 |  |  | 40-44 |  | 50-54 | 55-59 |  |  |  |  |
| Belgium | Males | high | 1952-1960 | 35-39 | 50-54 | 3 |  | 35-39 |  | 45-49 | 50-54 |  |  |  |  |  |
| Belgium | Females | low | 1927-1935 | 60-64 | 75-79 | 3 |  |  |  |  |  |  | 60-64 |  | 70-74 | 75-79 |
| Belgium | Females | low | 1932-1940 | 55-59 | 70-74 | 3 |  |  |  |  |  | 55-59 |  | 65-69 | 70-74 |  |
| Belgium | Females | low | 1937-1945 | 50-54 | 65-69 | 3 |  |  |  |  | 50-54 |  | 60-64 | 65-69 |  |  |
| Belgium | Females | low | 1942-1950 | 45-49 | 60-64 | 3 |  |  |  | 45-49 |  | 55-59 | 60-64 |  |  |  |
| Belgium | Females | low | 1947-1955 | 40-44 | 55-59 | 3 |  |  | 40-44 |  | 50-54 | 55-59 |  |  |  |  |
| Belgium | Females | low | 1952-1960 | 35-39 | 50-54 | 3 |  | 35-39 |  | 45-49 | 50-54 |  |  |  |  |  |
| Belgium | Females | high | 1927-1935 | 60-64 | 75-79 | 3 |  |  |  |  |  |  | 60-64 |  | 70-74 | 75-79 |
| Belgium | Females | high | 1932-1940 | 55-59 | 70-74 | 3 |  |  |  |  |  | 55-59 |  | 65-69 | 70-74 |  |
| Belgium | Females | high | 1937-1945 | 50-54 | 65-69 | 3 |  |  |  |  | 50-54 |  | 60-64 | 65-69 |  |  |
| Belgium | Females | high | 1942-1950 | 45-49 | 60-64 | 3 |  |  |  | 45-49 |  | 55-59 | 60-64 |  |  |  |
| Belgium | Females | high | 1947-1955 | 40-44 | 55-59 | 3 |  |  | 40-44 |  | 50-54 | 55-59 |  |  |  |  |
| Denmark | Males | low | 1922-1930 | 65-69 | 75-79 | 3 |  |  |  |  |  |  |  | 65-69 | 70-74 | 75-79 |
| Denmark | Males | low | 1927-1935 | 60-64 | 75-79 | 4 |  |  |  |  |  |  | 60-64 | 65-69 | 70-74 | 75-79 |
| Denmark | Males | low | 1932-1940 | 55-59 | 75-79 | 5 |  |  |  |  |  | 55-59 | 60-64 | 65-69 | 70-74 | 75-79 |
| Denmark | Males | low | 1937-1945 | 50-54 | 70-74 | 5 |  |  |  |  | 50-54 | 55-59 | 60-64 | 65-69 | 70-74 |  |
| Denmark | Males | low | 1942-1950 | 45-49 | 65-69 | 5 |  |  |  | 45-49 | 50-54 | 55-59 | 60-64 | 65-69 |  |  |
| Denmark | Males | low | 1947-1955 | 40-44 | 60-64 | 5 |  |  | 40-44 | 45-49 | 50-54 | 55-59 | 60-64 |  |  |  |
| Denmark | Males | low | 1952-1960 | 35-39 | 55-59 | 5 |  | 35-39 | 40-44 | 45-49 | 50-54 | 55-59 |  |  |  |  |
| Denmark | Males | low | 1957-1965 | 35-39 | 50-54 | 4 |  | 35-39 | 40-44 | 45-49 | 50-54 |  |  |  |  |  |
| Denmark | Males | high | 1922-1930 | 65-69 | 75-79 | 3 |  |  |  |  |  |  |  | 65-69 | 70-74 | 75-79 |
| Denmark | Males | high | 1927-1935 | 60-64 | 75-79 | 4 |  |  |  |  |  |  | 60-64 | 65-69 | 70-74 | 75-79 |
| Denmark | Males | high | 1932-1940 | 55-59 | 75-79 | 5 |  |  |  |  |  | 55-59 | 60-64 | 65-69 | 70-74 | 75-79 |
| Denmark | Males | high | 1937-1945 | 50-54 | 70-74 | 5 |  |  |  |  | 50-54 | 55-59 | 60-64 | 65-69 | 70-74 |  |
| Denmark | Males | high | 1942-1950 | 45-49 | 65-69 | 5 |  |  |  | 45-49 | 50-54 | 55-59 | 60-64 | 65-69 |  |  |
| Denmark | Males | high | 1947-1955 | 40-44 | 60-64 | 5 |  |  | 40-44 | 45-49 | 50-54 | 55-59 | 60-64 |  |  |  |
| Denmark | Males | high | 1952-1960 | 40-44 | 55-59 | 4 |  |  | 40-44 | 45-49 | 50-54 | 55-59 |  |  |  |  |
| Denmark | Females | low | 1922-1930 | 65-69 | 75-79 | 3 |  |  |  |  |  |  |  | 65-69 | 70-74 | 75-79 |
| Denmark | Females | low | 1927-1935 | 60-64 | 75-79 | 4 |  |  |  |  |  |  | 60-64 | 65-69 | 70-74 | 75-79 |
| Denmark | Females | low | 1932-1940 | 55-59 | 75-79 | 5 |  |  |  |  |  | 55-59 | 60-64 | 65-69 | 70-74 | 75-79 |
| Denmark | Females | low | 1937-1945 | 50-54 | 70-74 | 5 |  |  |  |  | 50-54 | 55-59 | 60-64 | 65-69 | 70-74 |  |
| Denmark | Females | low | 1942-1950 | 45-49 | 65-69 | 5 |  |  |  | 45-49 | 50-54 | 55-59 | 60-64 | 65-69 |  |  |
| Denmark | Females | low | 1947-1955 | 40-44 | 60-64 | 5 |  |  | 40-44 | 45-49 | 50-54 | 55-59 | 60-64 |  |  |  |
| Denmark | Females | low | 1952-1960 | 35-39 | 55-59 | 5 |  | 35-39 | 40-44 | 45-49 | 50-54 | 55-59 |  |  |  |  |
| Denmark | Females | low | 1957-1965 | 35-39 | 50-54 | 4 |  | 35-39 | 40-44 | 45-49 | 50-54 |  |  |  |  |  |
| Denmark | Females | low | 1962-1970 | 35-39 | 45-49 | 3 |  | 35-39 | 40-44 | 45-49 |  |  |  |  |  |  |
| Denmark | Females | high | 1922-1930 | 65-69 | 75-79 | 3 |  |  |  |  |  |  |  | 65-69 | 70-74 | 75-79 |
| Denmark | Females | high | 1927-1935 | 60-64 | 75-79 | 4 |  |  |  |  |  |  | 60-64 | 65-69 | 70-74 | 75-79 |
| Denmark | Females | high | 1932-1940 | 55-59 | 75-79 | 5 |  |  |  |  |  | 55-59 | 60-64 | 65-69 | 70-74 | 75-79 |
| Denmark | Females | high | 1937-1945 | 50-54 | 70-74 | 5 |  |  |  |  | 50-54 | 55-59 | 60-64 | 65-69 | 70-74 |  |
| Denmark | Females | high | 1942-1950 | 45-49 | 65-69 | 5 |  |  |  | 45-49 | 50-54 | 55-59 | 60-64 | 65-69 |  |  |
| Denmark | Females | high | 1947-1955 | 40-44 | 60-64 | 5 |  |  | 40-44 | 45-49 | 50-54 | 55-59 | 60-64 |  |  |  |
| Denmark | Females | high | 1952-1960 | 40-44 | 55-59 | 4 |  |  | 40-44 | 45-49 | 50-54 | 55-59 |  |  |  |  |
| Denmark | Females | high | 1957-1965 | 40-44 | 50-54 | 3 |  |  | 40-44 | 45-49 | 50-54 |  |  |  |  |  |
| Estonia | Males | low | 1922-1930 | 60-64 | 75-79 | 3 |  |  |  |  |  |  | 60-64 |  | 70-74 | 75-79 |
| Estonia | Males | low | 1927-1935 | 55-59 | 75-79 | 4 |  |  |  |  |  | 55-59 |  | 65-69 | 70-74 | 75-79 |
| Estonia | Males | low | 1932-1940 | 50-54 | 75-79 | 5 |  |  |  |  | 50-54 |  | 60-64 | 65-69 | 70-74 | 75-79 |
| Estonia | Males | low | 1937-1945 | 45-49 | 70-74 | 5 |  |  |  | 45-49 |  | 55-59 | 60-64 | 65-69 | 70-74 |  |
| Estonia | Males | low | 1942-1950 | 40-44 | 65-69 | 5 |  |  | 40-44 |  | 50-54 | 55-59 | 60-64 | 65-69 |  |  |
| Estonia | Males | low | 1947-1955 | 35-39 | 60-64 | 5 |  | 35-39 |  | 45-49 | 50-54 | 55-59 | 60-64 |  |  |  |
| Estonia | Males | low | 1952-1960 | 40-44 | 55-59 | 4 |  |  | 40-44 | 45-49 | 50-54 | 55-59 |  |  |  |  |
| Estonia | Males | high | 1922-1930 | 60-64 | 75-79 | 3 |  |  |  |  |  |  | 60-64 |  | 70-74 | 75-79 |
| Estonia | Males | high | 1927-1935 | 55-59 | 75-79 | 4 |  |  |  |  |  | 55-59 |  | 65-69 | 70-74 | 75-79 |
| Estonia | Males | high | 1932-1940 | 50-54 | 75-79 | 5 |  |  |  |  | 50-54 |  | 60-64 | 65-69 | 70-74 | 75-79 |
| Estonia | Males | high | 1937-1945 | 45-49 | 70-74 | 5 |  |  |  | 45-49 |  | 55-59 | 60-64 | 65-69 | 70-74 |  |
| Estonia | Males | high | 1942-1950 | 50-54 | 65-69 | 4 |  |  |  |  | 50-54 | 55-59 | 60-64 | 65-69 |  |  |
| Estonia | Males | high | 1947-1955 | 50-54 | 60-64 | 3 |  |  |  |  | 50-54 | 55-59 | 60-64 |  |  |  |
| Estonia | Females | low | 1922-1930 | 60-64 | 75-79 | 3 |  |  |  |  |  |  | 60-64 |  | 70-74 | 75-79 |
| Estonia | Females | low | 1927-1935 | 55-59 | 75-79 | 4 |  |  |  |  |  | 55-59 |  | 65-69 | 70-74 | 75-79 |
| Estonia | Females | low | 1932-1940 | 50-54 | 75-79 | 5 |  |  |  |  | 50-54 |  | 60-64 | 65-69 | 70-74 | 75-79 |
| Estonia | Females | low | 1937-1945 | 55-59 | 70-74 | 4 |  |  |  |  |  | 55-59 | 60-64 | 65-69 | 70-74 |  |
| Estonia | Females | low | 1942-1950 | 55-59 | 65-69 | 3 |  |  |  |  |  | 55-59 | 60-64 | 65-69 |  |  |
| Estonia | Females | low | 1947-1955 | 50-54 | 60-64 | 3 |  |  |  |  | 50-54 | 55-59 | 60-64 |  |  |  |
| Finland | Males | low | 1902-1910 | 65-69 | 75-79 | 3 |  |  |  |  |  |  |  | 65-69 | 70-74 | 75-79 |
| Finland | Males | low | 1907-1915 | 60-64 | 75-79 | 4 |  |  |  |  |  |  | 60-64 | 65-69 | 70-74 | 75-79 |
| Finland | Males | low | 1912-1920 | 55-59 | 75-79 | 5 |  |  |  |  |  | 55-59 | 60-64 | 65-69 | 70-74 | 75-79 |
| Finland | Males | low | 1917-1925 | 50-54 | 75-79 | 6 |  |  |  |  | 50-54 | 55-59 | 60-64 | 65-69 | 70-74 | 75-79 |
| Finland | Males | low | 1922-1930 | 45-49 | 75-79 | 7 |  |  |  | 45-49 | 50-54 | 55-59 | 60-64 | 65-69 | 70-74 | 75-79 |
| Finland | Males | low | 1927-1935 | 40-44 | 75-79 | 8 |  |  | 40-44 | 45-49 | 50-54 | 55-59 | 60-64 | 65-69 | 70-74 | 75-79 |
| Finland | Males | low | 1932-1940 | 35-39 | 75-79 | 9 |  | 35-39 | 40-44 | 45-49 | 50-54 | 55-59 | 60-64 | 65-69 | 70-74 | 75-79 |
| Finland | Males | low | 1937-1945 | 40-44 | 70-74 | 7 |  |  | 40-44 | 45-49 | 50-54 | 55-59 | 60-64 | 65-69 | 70-74 |  |
| Finland | Males | low | 1942-1950 | 35-39 | 65-69 | 7 |  | 35-39 | 40-44 | 45-49 | 50-54 | 55-59 | 60-64 | 65-69 |  |  |
| Finland | Males | low | 1947-1955 | 40-44 | 60-64 | 5 |  |  | 40-44 | 45-49 | 50-54 | 55-59 | 60-64 |  |  |  |
| Finland | Males | low | 1952-1960 | 35-39 | 55-59 | 5 |  | 35-39 | 40-44 | 45-49 | 50-54 | 55-59 |  |  |  |  |
| Finland | Males | low | 1957-1965 | 40-44 | 50-54 | 3 |  |  | 40-44 | 45-49 | 50-54 |  |  |  |  |  |
| Finland | Males | high | 1902-1910 | 65-69 | 75-79 | 3 |  |  |  |  |  |  |  | 65-69 | 70-74 | 75-79 |
| Finland | Males | high | 1907-1915 | 60-64 | 75-79 | 4 |  |  |  |  |  |  | 60-64 | 65-69 | 70-74 | 75-79 |
| Finland | Males | high | 1912-1920 | 55-59 | 75-79 | 5 |  |  |  |  |  | 55-59 | 60-64 | 65-69 | 70-74 | 75-79 |
| Finland | Males | high | 1917-1925 | 50-54 | 75-79 | 6 |  |  |  |  | 50-54 | 55-59 | 60-64 | 65-69 | 70-74 | 75-79 |
| Finland | Males | high | 1922-1930 | 50-54 | 75-79 | 6 |  |  |  |  | 50-54 | 55-59 | 60-64 | 65-69 | 70-74 | 75-79 |
| Finland | Males | high | 1927-1935 | 45-49 | 75-79 | 7 |  |  |  | 45-49 | 50-54 | 55-59 | 60-64 | 65-69 | 70-74 | 75-79 |
| Finland | Males | high | 1932-1940 | 50-54 | 75-79 | 6 |  |  |  |  | 50-54 | 55-59 | 60-64 | 65-69 | 70-74 | 75-79 |
| Finland | Males | high | 1937-1945 | 45-49 | 70-74 | 6 |  |  |  | 45-49 | 50-54 | 55-59 | 60-64 | 65-69 | 70-74 |  |
| Finland | Males | high | 1942-1950 | 45-49 | 65-69 | 5 |  |  |  | 45-49 | 50-54 | 55-59 | 60-64 | 65-69 |  |  |
| Finland | Males | high | 1947-1955 | 40-44 | 60-64 | 4 |  |  | 40-44 |  | 50-54 | 55-59 | 60-64 |  |  |  |
| Finland | Males | high | 1952-1960 | 45-49 | 55-59 | 3 |  |  |  | 45-49 | 50-54 | 55-59 |  |  |  |  |
| Finland | Females | low | 1902-1910 | 65-69 | 75-79 | 3 |  |  |  |  |  |  |  | 65-69 | 70-74 | 75-79 |
| Finland | Females | low | 1907-1915 | 60-64 | 75-79 | 4 |  |  |  |  |  |  | 60-64 | 65-69 | 70-74 | 75-79 |
| Finland | Females | low | 1912-1920 | 55-59 | 75-79 | 5 |  |  |  |  |  | 55-59 | 60-64 | 65-69 | 70-74 | 75-79 |
| Finland | Females | low | 1917-1925 | 50-54 | 75-79 | 6 |  |  |  |  | 50-54 | 55-59 | 60-64 | 65-69 | 70-74 | 75-79 |
| Finland | Females | low | 1922-1930 | 45-49 | 75-79 | 7 |  |  |  | 45-49 | 50-54 | 55-59 | 60-64 | 65-69 | 70-74 | 75-79 |
| Finland | Females | low | 1927-1935 | 40-44 | 75-79 | 8 |  |  | 40-44 | 45-49 | 50-54 | 55-59 | 60-64 | 65-69 | 70-74 | 75-79 |
| Finland | Females | low | 1932-1940 | 45-49 | 75-79 | 7 |  |  |  | 45-49 | 50-54 | 55-59 | 60-64 | 65-69 | 70-74 | 75-79 |
| Finland | Females | low | 1937-1945 | 45-49 | 70-74 | 6 |  |  |  | 45-49 | 50-54 | 55-59 | 60-64 | 65-69 | 70-74 |  |
| Finland | Females | low | 1942-1950 | 35-39 | 65-69 | 7 |  | 35-39 | 40-44 | 45-49 | 50-54 | 55-59 | 60-64 | 65-69 |  |  |
| Finland | Females | low | 1947-1955 | 40-44 | 60-64 | 5 |  |  | 40-44 | 45-49 | 50-54 | 55-59 | 60-64 |  |  |  |
| Finland | Females | low | 1952-1960 | 45-49 | 55-59 | 3 |  |  |  | 45-49 | 50-54 | 55-59 |  |  |  |  |
| Finland | Females | high | 1907-1915 | 65-69 | 75-79 | 3 |  |  |  |  |  |  |  | 65-69 | 70-74 | 75-79 |
| Finland | Females | high | 1912-1920 | 65-69 | 75-79 | 3 |  |  |  |  |  |  |  | 65-69 | 70-74 | 75-79 |
| Finland | Females | high | 1917-1925 | 65-69 | 75-79 | 3 |  |  |  |  |  |  |  | 65-69 | 70-74 | 75-79 |
| Finland | Females | high | 1922-1930 | 60-64 | 75-79 | 4 |  |  |  |  |  |  | 60-64 | 65-69 | 70-74 | 75-79 |
| Finland | Females | high | 1927-1935 | 60-64 | 75-79 | 4 |  |  |  |  |  |  | 60-64 | 65-69 | 70-74 | 75-79 |
| Finland | Females | high | 1932-1940 | 60-64 | 75-79 | 4 |  |  |  |  |  |  | 60-64 | 65-69 | 70-74 | 75-79 |
| Finland | Females | high | 1937-1945 | 50-54 | 70-74 | 5 |  |  |  |  | 50-54 | 55-59 | 60-64 | 65-69 | 70-74 |  |
| Finland | Females | high | 1942-1950 | 45-49 | 65-69 | 5 |  |  |  | 45-49 | 50-54 | 55-59 | 60-64 | 65-69 |  |  |
| Finland | Females | high | 1947-1955 | 40-44 | 60-64 | 5 |  |  | 40-44 | 45-49 | 50-54 | 55-59 | 60-64 |  |  |  |
| Finland | Females | high | 1952-1960 | 45-49 | 55-59 | 3 |  |  |  | 45-49 | 50-54 | 55-59 |  |  |  |  |
| Hungary | Males | low | 1907-1915 | 60-64 | 75-79 | 3 |  |  |  |  |  |  | 60-64 | 65-69 |  | 75-79 |
| Hungary | Males | low | 1912-1920 | 55-59 | 70-74 | 3 |  |  |  |  |  | 55-59 | 60-64 |  | 70-74 |  |
| Hungary | Males | low | 1917-1925 | 50-54 | 75-79 | 4 |  |  |  |  | 50-54 | 55-59 |  | 65-69 |  | 75-79 |
| Hungary | Males | low | 1922-1930 | 45-49 | 70-74 | 4 |  |  |  | 45-49 | 50-54 |  | 60-64 |  | 70-74 |  |
| Hungary | Males | low | 1927-1935 | 40-44 | 75-79 | 5 |  |  | 40-44 | 45-49 |  | 55-59 |  | 65-69 |  | 75-79 |
| Hungary | Males | low | 1932-1940 | 35-39 | 70-74 | 5 |  | 35-39 | 40-44 |  | 50-54 |  | 60-64 |  | 70-74 |  |
| Hungary | Males | low | 1937-1945 | 30-34 | 65-69 | 5 | 30-34 | 35-39 |  | 45-49 |  | 55-59 |  | 65-69 |  |  |
| Hungary | Males | low | 1942-1950 | 30-34 | 60-64 | 4 | 30-34 |  | 40-44 |  | 50-54 |  | 60-64 |  |  |  |
| Hungary | Males | low | 1947-1955 | 35-39 | 55-59 | 3 |  | 35-39 |  | 45-49 |  | 55-59 |  |  |  |  |
| Hungary | Males | low | 1952-1960 | 30-34 | 50-54 | 3 | 30-34 |  | 40-44 |  | 50-54 |  |  |  |  |  |
| Hungary | Males | high | 1907-1915 | 60-64 | 75-79 | 3 |  |  |  |  |  |  | 60-64 | 65-69 |  | 75-79 |
| Hungary | Males | high | 1912-1920 | 55-59 | 70-74 | 3 |  |  |  |  |  | 55-59 | 60-64 |  | 70-74 |  |
| Hungary | Males | high | 1917-1925 | 50-54 | 75-79 | 4 |  |  |  |  | 50-54 | 55-59 |  | 65-69 |  | 75-79 |
| Hungary | Males | high | 1922-1930 | 45-49 | 70-74 | 4 |  |  |  | 45-49 | 50-54 |  | 60-64 |  | 70-74 |  |
| Hungary | Males | high | 1927-1935 | 40-44 | 75-79 | 5 |  |  | 40-44 | 45-49 |  | 55-59 |  | 65-69 |  | 75-79 |
| Hungary | Males | high | 1932-1940 | 40-44 | 70-74 | 4 |  |  | 40-44 |  | 50-54 |  | 60-64 |  | 70-74 |  |
| Hungary | Males | high | 1937-1945 | 45-49 | 65-69 | 3 |  |  |  | 45-49 |  | 55-59 |  | 65-69 |  |  |
| Hungary | Males | high | 1942-1950 | 40-44 | 60-64 | 3 |  |  | 40-44 |  | 50-54 |  | 60-64 |  |  |  |
| Hungary | Males | high | 1947-1955 | 35-39 | 55-59 | 3 |  | 35-39 |  | 45-49 |  | 55-59 |  |  |  |  |
| Hungary | Females | low | 1907-1915 | 60-64 | 75-79 | 3 |  |  |  |  |  |  | 60-64 | 65-69 |  | 75-79 |
| Hungary | Females | low | 1912-1920 | 55-59 | 70-74 | 3 |  |  |  |  |  | 55-59 | 60-64 |  | 70-74 |  |
| Hungary | Females | low | 1917-1925 | 50-54 | 75-79 | 4 |  |  |  |  | 50-54 | 55-59 |  | 65-69 |  | 75-79 |
| Hungary | Females | low | 1922-1930 | 45-49 | 70-74 | 4 |  |  |  | 45-49 | 50-54 |  | 60-64 |  | 70-74 |  |
| Hungary | Females | low | 1927-1935 | 40-44 | 75-79 | 5 |  |  | 40-44 | 45-49 |  | 55-59 |  | 65-69 |  | 75-79 |
| Hungary | Females | low | 1932-1940 | 35-39 | 70-74 | 5 |  | 35-39 | 40-44 |  | 50-54 |  | 60-64 |  | 70-74 |  |
| Hungary | Females | low | 1937-1945 | 30-34 | 65-69 | 5 | 30-34 | 35-39 |  | 45-49 |  | 55-59 |  | 65-69 |  |  |
| Hungary | Females | low | 1942-1950 | 30-34 | 60-64 | 4 | 30-34 |  | 40-44 |  | 50-54 |  | 60-64 |  |  |  |
| Hungary | Females | low | 1947-1955 | 35-39 | 55-59 | 3 |  | 35-39 |  | 45-49 |  | 55-59 |  |  |  |  |
| Hungary | Females | low | 1952-1960 | 30-34 | 50-54 | 3 | 30-34 |  | 40-44 |  | 50-54 |  |  |  |  |  |
| Hungary | Females | high | 1907-1915 | 60-64 | 75-79 | 3 |  |  |  |  |  |  | 60-64 | 65-69 |  | 75-79 |
| Hungary | Females | high | 1917-1925 | 55-59 | 75-79 | 3 |  |  |  |  |  | 55-59 |  | 65-69 |  | 75-79 |
| Hungary | Females | high | 1922-1930 | 50-54 | 70-74 | 3 |  |  |  |  | 50-54 |  | 60-64 |  | 70-74 |  |
| Hungary | Females | high | 1927-1935 | 45-49 | 75-79 | 4 |  |  |  | 45-49 |  | 55-59 |  | 65-69 |  | 75-79 |
| Hungary | Females | high | 1932-1940 | 50-54 | 70-74 | 3 |  |  |  |  | 50-54 |  | 60-64 |  | 70-74 |  |
| Hungary | Females | high | 1937-1945 | 45-49 | 65-69 | 3 |  |  |  | 45-49 |  | 55-59 |  | 65-69 |  |  |
| Hungary | Females | high | 1942-1950 | 40-44 | 60-64 | 3 |  |  | 40-44 |  | 50-54 |  | 60-64 |  |  |  |
| Italy (T) | Males | low | 1902-1910 | 65-69 | 75-79 | 3 |  |  |  |  |  |  |  | 65-69 | 70-74 | 75-79 |
| Italy (T) | Males | low | 1907-1915 | 60-64 | 75-79 | 4 |  |  |  |  |  |  | 60-64 | 65-69 | 70-74 | 75-79 |
| Italy (T) | Males | low | 1912-1920 | 55-59 | 75-79 | 5 |  |  |  |  |  | 55-59 | 60-64 | 65-69 | 70-74 | 75-79 |
| Italy (T) | Males | low | 1917-1925 | 50-54 | 75-79 | 6 |  |  |  |  | 50-54 | 55-59 | 60-64 | 65-69 | 70-74 | 75-79 |
| Italy (T) | Males | low | 1922-1930 | 45-49 | 75-79 | 7 |  |  |  | 45-49 | 50-54 | 55-59 | 60-64 | 65-69 | 70-74 | 75-79 |
| Italy (T) | Males | low | 1927-1935 | 40-44 | 75-79 | 8 |  |  | 40-44 | 45-49 | 50-54 | 55-59 | 60-64 | 65-69 | 70-74 | 75-79 |
| Italy (T) | Males | low | 1932-1940 | 35-39 | 75-79 | 9 |  | 35-39 | 40-44 | 45-49 | 50-54 | 55-59 | 60-64 | 65-69 | 70-74 | 75-79 |
| Italy (T) | Males | low | 1937-1945 | 40-44 | 70-74 | 7 |  |  | 40-44 | 45-49 | 50-54 | 55-59 | 60-64 | 65-69 | 70-74 |  |
| Italy (T) | Males | low | 1942-1950 | 40-44 | 65-69 | 6 |  |  | 40-44 | 45-49 | 50-54 | 55-59 | 60-64 | 65-69 |  |  |
| Italy (T) | Males | low | 1947-1955 | 40-44 | 60-64 | 5 |  |  | 40-44 | 45-49 | 50-54 | 55-59 | 60-64 |  |  |  |
| Italy (T) | Males | low | 1952-1960 | 45-49 | 55-59 | 3 |  |  |  | 45-49 | 50-54 | 55-59 |  |  |  |  |
| Italy (T) | Males | high | 1907-1915 | 65-69 | 75-79 | 3 |  |  |  |  |  |  |  | 65-69 | 70-74 | 75-79 |
| Italy (T) | Males | high | 1912-1920 | 65-69 | 75-79 | 3 |  |  |  |  |  |  |  | 65-69 | 70-74 | 75-79 |
| Italy (T) | Males | high | 1917-1925 | 60-64 | 75-79 | 4 |  |  |  |  |  |  | 60-64 | 65-69 | 70-74 | 75-79 |
| Italy (T) | Males | high | 1922-1930 | 55-59 | 75-79 | 4 |  |  |  |  |  | 55-59 |  | 65-69 | 70-74 | 75-79 |
| Italy (T) | Males | high | 1927-1935 | 60-64 | 75-79 | 4 |  |  |  |  |  |  | 60-64 | 65-69 | 70-74 | 75-79 |
| Italy (T) | Males | high | 1932-1940 | 60-64 | 75-79 | 4 |  |  |  |  |  |  | 60-64 | 65-69 | 70-74 | 75-79 |
| Italy (T) | Males | high | 1937-1945 | 60-64 | 70-74 | 3 |  |  |  |  |  |  | 60-64 | 65-69 | 70-74 |  |
| Italy (T) | Males | high | 1942-1950 | 50-54 | 65-69 | 4 |  |  |  |  | 50-54 | 55-59 | 60-64 | 65-69 |  |  |
| Italy (T) | Females | low | 1902-1910 | 65-69 | 75-79 | 3 |  |  |  |  |  |  |  | 65-69 | 70-74 | 75-79 |
| Italy (T) | Females | low | 1907-1915 | 60-64 | 75-79 | 4 |  |  |  |  |  |  | 60-64 | 65-69 | 70-74 | 75-79 |
| Italy (T) | Females | low | 1912-1920 | 55-59 | 75-79 | 5 |  |  |  |  |  | 55-59 | 60-64 | 65-69 | 70-74 | 75-79 |
| Italy (T) | Females | low | 1917-1925 | 50-54 | 75-79 | 6 |  |  |  |  | 50-54 | 55-59 | 60-64 | 65-69 | 70-74 | 75-79 |
| Italy (T) | Females | low | 1922-1930 | 50-54 | 75-79 | 6 |  |  |  |  | 50-54 | 55-59 | 60-64 | 65-69 | 70-74 | 75-79 |
| Italy (T) | Females | low | 1927-1935 | 50-54 | 75-79 | 6 |  |  |  |  | 50-54 | 55-59 | 60-64 | 65-69 | 70-74 | 75-79 |
| Italy (T) | Females | low | 1932-1940 | 45-49 | 75-79 | 7 |  |  |  | 45-49 | 50-54 | 55-59 | 60-64 | 65-69 | 70-74 | 75-79 |
| Italy (T) | Females | low | 1937-1945 | 40-44 | 70-74 | 7 |  |  | 40-44 | 45-49 | 50-54 | 55-59 | 60-64 | 65-69 | 70-74 |  |
| Italy (T) | Females | low | 1942-1950 | 45-49 | 65-69 | 5 |  |  |  | 45-49 | 50-54 | 55-59 | 60-64 | 65-69 |  |  |
| Italy (T) | Females | low | 1947-1955 | 50-54 | 60-64 | 3 |  |  |  |  | 50-54 | 55-59 | 60-64 |  |  |  |
| Lithuania | Males | low | 1927-1935 | 55-59 | 75-79 | 3 |  |  |  |  |  | 55-59 |  |  | 70-74 | 75-79 |
| Lithuania | Males | low | 1932-1940 | 50-54 | 75-79 | 4 |  |  |  |  | 50-54 |  |  | 65-69 | 70-74 | 75-79 |
| Lithuania | Males | low | 1937-1945 | 45-49 | 70-74 | 4 |  |  |  | 45-49 |  |  | 60-64 | 65-69 | 70-74 |  |
| Lithuania | Males | low | 1942-1950 | 40-44 | 65-69 | 4 |  |  | 40-44 |  |  | 55-59 | 60-64 | 65-69 |  |  |
| Lithuania | Males | low | 1947-1955 | 35-39 | 60-64 | 4 |  | 35-39 |  |  | 50-54 | 55-59 | 60-64 |  |  |  |
| Lithuania | Males | low | 1952-1960 | 45-49 | 55-59 | 3 |  |  |  | 45-49 | 50-54 | 55-59 |  |  |  |  |
| Lithuania | Males | high | 1932-1940 | 65-69 | 75-79 | 3 |  |  |  |  |  |  |  | 65-69 | 70-74 | 75-79 |
| Lithuania | Males | high | 1937-1945 | 60-64 | 70-74 | 3 |  |  |  |  |  |  | 60-64 | 65-69 | 70-74 |  |
| Lithuania | Males | high | 1942-1950 | 55-59 | 65-69 | 3 |  |  |  |  |  | 55-59 | 60-64 | 65-69 |  |  |
| Lithuania | Males | high | 1947-1955 | 50-54 | 60-64 | 3 |  |  |  |  | 50-54 | 55-59 | 60-64 |  |  |  |
| Lithuania | Males | high | 1952-1960 | 45-49 | 55-59 | 3 |  |  |  | 45-49 | 50-54 | 55-59 |  |  |  |  |
| Lithuania | Females | low | 1927-1935 | 55-59 | 75-79 | 3 |  |  |  |  |  | 55-59 |  |  | 70-74 | 75-79 |
| Lithuania | Females | low | 1932-1940 | 50-54 | 75-79 | 4 |  |  |  |  | 50-54 |  |  | 65-69 | 70-74 | 75-79 |
| Lithuania | Females | low | 1937-1945 | 60-64 | 70-74 | 3 |  |  |  |  |  |  | 60-64 | 65-69 | 70-74 |  |
| Lithuania | Females | low | 1942-1950 | 55-59 | 65-69 | 3 |  |  |  |  |  | 55-59 | 60-64 | 65-69 |  |  |
| Lithuania | Females | low | 1947-1955 | 50-54 | 60-64 | 3 |  |  |  |  | 50-54 | 55-59 | 60-64 |  |  |  |
| Lithuania | Females | high | 1932-1940 | 65-69 | 75-79 | 3 |  |  |  |  |  |  |  | 65-69 | 70-74 | 75-79 |
| Lithuania | Females | high | 1937-1945 | 60-64 | 70-74 | 3 |  |  |  |  |  |  | 60-64 | 65-69 | 70-74 |  |
| Lithuania | Females | high | 1942-1950 | 55-59 | 65-69 | 3 |  |  |  |  |  | 55-59 | 60-64 | 65-69 |  |  |
| Norway | Males | low | 1902-1910 | 65-69 | 75-79 | 3 |  |  |  |  |  |  |  | 65-69 | 70-74 | 75-79 |
| Norway | Males | low | 1907-1915 | 60-64 | 75-79 | 4 |  |  |  |  |  |  | 60-64 | 65-69 | 70-74 | 75-79 |
| Norway | Males | low | 1912-1920 | 55-59 | 75-79 | 5 |  |  |  |  |  | 55-59 | 60-64 | 65-69 | 70-74 | 75-79 |
| Norway | Males | low | 1917-1925 | 50-54 | 75-79 | 6 |  |  |  |  | 50-54 | 55-59 | 60-64 | 65-69 | 70-74 | 75-79 |
| Norway | Males | low | 1922-1930 | 45-49 | 75-79 | 7 |  |  |  | 45-49 | 50-54 | 55-59 | 60-64 | 65-69 | 70-74 | 75-79 |
| Norway | Males | low | 1927-1935 | 40-44 | 75-79 | 8 |  |  | 40-44 | 45-49 | 50-54 | 55-59 | 60-64 | 65-69 | 70-74 | 75-79 |
| Norway | Males | low | 1932-1940 | 40-44 | 70-74 | 7 |  |  | 40-44 | 45-49 | 50-54 | 55-59 | 60-64 | 65-69 | 70-74 |  |
| Norway | Males | low | 1937-1945 | 40-44 | 65-69 | 6 |  |  | 40-44 | 45-49 | 50-54 | 55-59 | 60-64 | 65-69 |  |  |
| Norway | Males | low | 1942-1950 | 40-44 | 60-64 | 5 |  |  | 40-44 | 45-49 | 50-54 | 55-59 | 60-64 |  |  |  |
| Norway | Males | low | 1947-1955 | 40-44 | 55-59 | 4 |  |  | 40-44 | 45-49 | 50-54 | 55-59 |  |  |  |  |
| Norway | Males | low | 1952-1960 | 40-44 | 50-54 | 3 |  |  | 40-44 | 45-49 | 50-54 |  |  |  |  |  |
| Norway | Males | high | 1902-1910 | 65-69 | 75-79 | 3 |  |  |  |  |  |  |  | 65-69 | 70-74 | 75-79 |
| Norway | Males | high | 1907-1915 | 60-64 | 75-79 | 4 |  |  |  |  |  |  | 60-64 | 65-69 | 70-74 | 75-79 |
| Norway | Males | high | 1912-1920 | 55-59 | 75-79 | 5 |  |  |  |  |  | 55-59 | 60-64 | 65-69 | 70-74 | 75-79 |
| Norway | Males | high | 1917-1925 | 50-54 | 75-79 | 6 |  |  |  |  | 50-54 | 55-59 | 60-64 | 65-69 | 70-74 | 75-79 |
| Norway | Males | high | 1922-1930 | 50-54 | 75-79 | 6 |  |  |  |  | 50-54 | 55-59 | 60-64 | 65-69 | 70-74 | 75-79 |
| Norway | Males | high | 1927-1935 | 50-54 | 75-79 | 6 |  |  |  |  | 50-54 | 55-59 | 60-64 | 65-69 | 70-74 | 75-79 |
| Norway | Males | high | 1932-1940 | 50-54 | 70-74 | 5 |  |  |  |  | 50-54 | 55-59 | 60-64 | 65-69 | 70-74 |  |
| Norway | Males | high | 1937-1945 | 50-54 | 65-69 | 4 |  |  |  |  | 50-54 | 55-59 | 60-64 | 65-69 |  |  |
| Norway | Males | high | 1942-1950 | 50-54 | 60-64 | 3 |  |  |  |  | 50-54 | 55-59 | 60-64 |  |  |  |
| Norway | Females | low | 1902-1910 | 65-69 | 75-79 | 3 |  |  |  |  |  |  |  | 65-69 | 70-74 | 75-79 |
| Norway | Females | low | 1907-1915 | 60-64 | 75-79 | 4 |  |  |  |  |  |  | 60-64 | 65-69 | 70-74 | 75-79 |
| Norway | Females | low | 1912-1920 | 55-59 | 75-79 | 5 |  |  |  |  |  | 55-59 | 60-64 | 65-69 | 70-74 | 75-79 |
| Norway | Females | low | 1917-1925 | 50-54 | 75-79 | 6 |  |  |  |  | 50-54 | 55-59 | 60-64 | 65-69 | 70-74 | 75-79 |
| Norway | Females | low | 1922-1930 | 45-49 | 75-79 | 7 |  |  |  | 45-49 | 50-54 | 55-59 | 60-64 | 65-69 | 70-74 | 75-79 |
| Norway | Females | low | 1927-1935 | 45-49 | 75-79 | 7 |  |  |  | 45-49 | 50-54 | 55-59 | 60-64 | 65-69 | 70-74 | 75-79 |
| Norway | Females | low | 1932-1940 | 45-49 | 70-74 | 6 |  |  |  | 45-49 | 50-54 | 55-59 | 60-64 | 65-69 | 70-74 |  |
| Norway | Females | low | 1937-1945 | 40-44 | 65-69 | 5 |  |  | 40-44 |  | 50-54 | 55-59 | 60-64 | 65-69 |  |  |
| Norway | Females | low | 1942-1950 | 45-49 | 60-64 | 4 |  |  |  | 45-49 | 50-54 | 55-59 | 60-64 |  |  |  |
| Norway | Females | low | 1947-1955 | 40-44 | 55-59 | 4 |  |  | 40-44 | 45-49 | 50-54 | 55-59 |  |  |  |  |
| Norway | Females | low | 1952-1960 | 40-44 | 50-54 | 3 |  |  | 40-44 | 45-49 | 50-54 |  |  |  |  |  |
| Norway | Females | high | 1912-1920 | 65-69 | 75-79 | 3 |  |  |  |  |  |  |  | 65-69 | 70-74 | 75-79 |
| Norway | Females | high | 1917-1925 | 60-64 | 75-79 | 4 |  |  |  |  |  |  | 60-64 | 65-69 | 70-74 | 75-79 |
| Norway | Females | high | 1922-1930 | 65-69 | 75-79 | 3 |  |  |  |  |  |  |  | 65-69 | 70-74 | 75-79 |
| Norway | Females | high | 1927-1935 | 60-64 | 75-79 | 4 |  |  |  |  |  |  | 60-64 | 65-69 | 70-74 | 75-79 |
| Norway | Females | high | 1932-1940 | 60-64 | 70-74 | 3 |  |  |  |  |  |  | 60-64 | 65-69 | 70-74 |  |
| Norway | Females | high | 1937-1945 | 55-59 | 65-69 | 3 |  |  |  |  |  | 55-59 | 60-64 | 65-69 |  |  |
| Norway | Females | high | 1942-1950 | 50-54 | 60-64 | 3 |  |  |  |  | 50-54 | 55-59 | 60-64 |  |  |  |
| Spain (B) | Males | low | 1922-1930 | 65-69 | 75-79 | 3 |  |  |  |  |  |  |  | 65-69 | 70-74 | 75-79 |
| Spain (B) | Males | low | 1927-1935 | 60-64 | 75-79 | 4 |  |  |  |  |  |  | 60-64 | 65-69 | 70-74 | 75-79 |
| Spain (B) | Males | low | 1932-1940 | 55-59 | 75-79 | 5 |  |  |  |  |  | 55-59 | 60-64 | 65-69 | 70-74 | 75-79 |
| Spain (B) | Males | low | 1937-1945 | 50-54 | 70-74 | 5 |  |  |  |  | 50-54 | 55-59 | 60-64 | 65-69 | 70-74 |  |
| Spain (B) | Males | low | 1942-1950 | 45-49 | 65-69 | 5 |  |  |  | 45-49 | 50-54 | 55-59 | 60-64 | 65-69 |  |  |
| Spain (B) | Males | low | 1947-1955 | 40-44 | 60-64 | 5 |  |  | 40-44 | 45-49 | 50-54 | 55-59 | 60-64 |  |  |  |
| Spain (B) | Males | low | 1952-1960 | 35-39 | 55-59 | 5 |  | 35-39 | 40-44 | 45-49 | 50-54 | 55-59 |  |  |  |  |
| Spain (B) | Males | low | 1957-1965 | 35-39 | 50-54 | 4 |  | 35-39 | 40-44 | 45-49 | 50-54 |  |  |  |  |  |
| Spain (B) | Males | high | 1922-1930 | 65-69 | 75-79 | 3 |  |  |  |  |  |  |  | 65-69 | 70-74 | 75-79 |
| Spain (B) | Males | high | 1927-1935 | 60-64 | 75-79 | 4 |  |  |  |  |  |  | 60-64 | 65-69 | 70-74 | 75-79 |
| Spain (B) | Males | high | 1932-1940 | 55-59 | 75-79 | 5 |  |  |  |  |  | 55-59 | 60-64 | 65-69 | 70-74 | 75-79 |
| Spain (B) | Males | high | 1937-1945 | 50-54 | 70-74 | 5 |  |  |  |  | 50-54 | 55-59 | 60-64 | 65-69 | 70-74 |  |
| Spain (B) | Males | high | 1942-1950 | 45-49 | 65-69 | 5 |  |  |  | 45-49 | 50-54 | 55-59 | 60-64 | 65-69 |  |  |
| Spain (B) | Males | high | 1947-1955 | 40-44 | 60-64 | 5 |  |  | 40-44 | 45-49 | 50-54 | 55-59 | 60-64 |  |  |  |
| Spain (B) | Males | high | 1952-1960 | 45-49 | 55-59 | 3 |  |  |  | 45-49 | 50-54 | 55-59 |  |  |  |  |
| Spain (B) | Females | low | 1922-1930 | 65-69 | 75-79 | 3 |  |  |  |  |  |  |  | 65-69 | 70-74 | 75-79 |
| Spain (B) | Females | low | 1927-1935 | 60-64 | 75-79 | 4 |  |  |  |  |  |  | 60-64 | 65-69 | 70-74 | 75-79 |
| Spain (B) | Females | low | 1932-1940 | 55-59 | 75-79 | 5 |  |  |  |  |  | 55-59 | 60-64 | 65-69 | 70-74 | 75-79 |
| Spain (B) | Females | low | 1937-1945 | 55-59 | 70-74 | 4 |  |  |  |  |  | 55-59 | 60-64 | 65-69 | 70-74 |  |
| Spain (B) | Females | low | 1942-1950 | 45-49 | 65-69 | 5 |  |  |  | 45-49 | 50-54 | 55-59 | 60-64 | 65-69 |  |  |
| Spain (B) | Females | low | 1947-1955 | 45-49 | 60-64 | 4 |  |  |  | 45-49 | 50-54 | 55-59 | 60-64 |  |  |  |
| Spain (B) | Females | low | 1952-1960 | 40-44 | 55-59 | 4 |  |  | 40-44 | 45-49 | 50-54 | 55-59 |  |  |  |  |
| Spain (B) | Females | low | 1957-1965 | 40-44 | 50-54 | 3 |  |  | 40-44 | 45-49 | 50-54 |  |  |  |  |  |
| Spain (B) | Females | high | 1937-1945 | 60-64 | 70-74 | 3 |  |  |  |  |  |  | 60-64 | 65-69 | 70-74 |  |
| Spain (B) | Females | high | 1942-1950 | 55-59 | 65-69 | 3 |  |  |  |  |  | 55-59 | 60-64 | 65-69 |  |  |
| Spain (B) | Females | high | 1947-1955 | 50-54 | 60-64 | 3 |  |  |  |  | 50-54 | 55-59 | 60-64 |  |  |  |
| Spain (B) | Females | high | 1952-1960 | 45-49 | 55-59 | 3 |  |  |  | 45-49 | 50-54 | 55-59 |  |  |  |  |
| Sweden | Males | low | 1922-1930 | 65-69 | 75-79 | 3 |  |  |  |  |  |  |  | 65-69 | 70-74 | 75-79 |
| Sweden | Males | low | 1927-1935 | 60-64 | 75-79 | 4 |  |  |  |  |  |  | 60-64 | 65-69 | 70-74 | 75-79 |
| Sweden | Males | low | 1932-1940 | 55-59 | 70-74 | 4 |  |  |  |  |  | 55-59 | 60-64 | 65-69 | 70-74 |  |
| Sweden | Males | low | 1937-1945 | 50-54 | 65-69 | 4 |  |  |  |  | 50-54 | 55-59 | 60-64 | 65-69 |  |  |
| Sweden | Males | low | 1942-1950 | 45-49 | 60-64 | 4 |  |  |  | 45-49 | 50-54 | 55-59 | 60-64 |  |  |  |
| Sweden | Males | low | 1947-1955 | 40-44 | 55-59 | 4 |  |  | 40-44 | 45-49 | 50-54 | 55-59 |  |  |  |  |
| Sweden | Males | low | 1952-1960 | 40-44 | 50-54 | 3 |  |  | 40-44 | 45-49 | 50-54 |  |  |  |  |  |
| Sweden | Males | high | 1922-1930 | 65-69 | 75-79 | 3 |  |  |  |  |  |  |  | 65-69 | 70-74 | 75-79 |
| Sweden | Males | high | 1927-1935 | 60-64 | 75-79 | 4 |  |  |  |  |  |  | 60-64 | 65-69 | 70-74 | 75-79 |
| Sweden | Males | high | 1932-1940 | 55-59 | 70-74 | 4 |  |  |  |  |  | 55-59 | 60-64 | 65-69 | 70-74 |  |
| Sweden | Males | high | 1937-1945 | 50-54 | 65-69 | 4 |  |  |  |  | 50-54 | 55-59 | 60-64 | 65-69 |  |  |
| Sweden | Males | high | 1942-1950 | 45-49 | 60-64 | 4 |  |  |  | 45-49 | 50-54 | 55-59 | 60-64 |  |  |  |
| Sweden | Males | high | 1947-1955 | 45-49 | 55-59 | 3 |  |  |  | 45-49 | 50-54 | 55-59 |  |  |  |  |
| Sweden | Females | low | 1922-1930 | 65-69 | 75-79 | 3 |  |  |  |  |  |  |  | 65-69 | 70-74 | 75-79 |
| Sweden | Females | low | 1927-1935 | 60-64 | 75-79 | 4 |  |  |  |  |  |  | 60-64 | 65-69 | 70-74 | 75-79 |
| Sweden | Females | low | 1932-1940 | 55-59 | 70-74 | 4 |  |  |  |  |  | 55-59 | 60-64 | 65-69 | 70-74 |  |
| Sweden | Females | low | 1937-1945 | 50-54 | 65-69 | 4 |  |  |  |  | 50-54 | 55-59 | 60-64 | 65-69 |  |  |
| Sweden | Females | low | 1942-1950 | 45-49 | 60-64 | 4 |  |  |  | 45-49 | 50-54 | 55-59 | 60-64 |  |  |  |
| Sweden | Females | low | 1947-1955 | 40-44 | 55-59 | 4 |  |  | 40-44 | 45-49 | 50-54 | 55-59 |  |  |  |  |
| Sweden | Females | low | 1952-1960 | 40-44 | 50-54 | 3 |  |  | 40-44 | 45-49 | 50-54 |  |  |  |  |  |
| Sweden | Females | low | 1957-1965 | 35-39 | 45-49 | 3 |  | 35-39 | 40-44 | 45-49 |  |  |  |  |  |  |
| Sweden | Females | high | 1922-1930 | 65-69 | 75-79 | 3 |  |  |  |  |  |  |  | 65-69 | 70-74 | 75-79 |
| Sweden | Females | high | 1927-1935 | 60-64 | 75-79 | 4 |  |  |  |  |  |  | 60-64 | 65-69 | 70-74 | 75-79 |
| Sweden | Females | high | 1932-1940 | 55-59 | 70-74 | 4 |  |  |  |  |  | 55-59 | 60-64 | 65-69 | 70-74 |  |
| Sweden | Females | high | 1937-1945 | 50-54 | 65-69 | 4 |  |  |  |  | 50-54 | 55-59 | 60-64 | 65-69 |  |  |
| Sweden | Females | high | 1942-1950 | 45-49 | 60-64 | 4 |  |  |  | 45-49 | 50-54 | 55-59 | 60-64 |  |  |  |
| Sweden | Females | high | 1947-1955 | 40-44 | 55-59 | 4 |  |  | 40-44 | 45-49 | 50-54 | 55-59 |  |  |  |  |
| Sweden | Females | high | 1952-1960 | 40-44 | 50-54 | 3 |  |  | 40-44 | 45-49 | 50-54 |  |  |  |  |  |
| Switzerland | Males | low | 1922-1930 | 65-69 | 75-79 | 3 |  |  |  |  |  |  |  | 65-69 | 70-74 | 75-79 |
| Switzerland | Males | low | 1927-1935 | 60-64 | 75-79 | 4 |  |  |  |  |  |  | 60-64 | 65-69 | 70-74 | 75-79 |
| Switzerland | Males | low | 1932-1940 | 55-59 | 75-79 | 5 |  |  |  |  |  | 55-59 | 60-64 | 65-69 | 70-74 | 75-79 |
| Switzerland | Males | low | 1937-1945 | 50-54 | 70-74 | 5 |  |  |  |  | 50-54 | 55-59 | 60-64 | 65-69 | 70-74 |  |
| Switzerland | Males | low | 1942-1950 | 45-49 | 65-69 | 5 |  |  |  | 45-49 | 50-54 | 55-59 | 60-64 | 65-69 |  |  |
| Switzerland | Males | low | 1947-1955 | 40-44 | 60-64 | 5 |  |  | 40-44 | 45-49 | 50-54 | 55-59 | 60-64 |  |  |  |
| Switzerland | Males | low | 1952-1960 | 40-44 | 55-59 | 4 |  |  | 40-44 | 45-49 | 50-54 | 55-59 |  |  |  |  |
| Switzerland | Males | high | 1922-1930 | 65-69 | 75-79 | 3 |  |  |  |  |  |  |  | 65-69 | 70-74 | 75-79 |
| Switzerland | Males | high | 1927-1935 | 60-64 | 75-79 | 4 |  |  |  |  |  |  | 60-64 | 65-69 | 70-74 | 75-79 |
| Switzerland | Males | high | 1932-1940 | 55-59 | 75-79 | 5 |  |  |  |  |  | 55-59 | 60-64 | 65-69 | 70-74 | 75-79 |
| Switzerland | Males | high | 1937-1945 | 50-54 | 70-74 | 5 |  |  |  |  | 50-54 | 55-59 | 60-64 | 65-69 | 70-74 |  |
| Switzerland | Males | high | 1942-1950 | 45-49 | 65-69 | 5 |  |  |  | 45-49 | 50-54 | 55-59 | 60-64 | 65-69 |  |  |
| Switzerland | Males | high | 1947-1955 | 40-44 | 60-64 | 5 |  |  | 40-44 | 45-49 | 50-54 | 55-59 | 60-64 |  |  |  |
| Switzerland | Males | high | 1952-1960 | 45-49 | 55-59 | 3 |  |  |  | 45-49 | 50-54 | 55-59 |  |  |  |  |
| Switzerland | Males | high | 1957-1965 | 40-44 | 50-54 | 3 |  |  | 40-44 | 45-49 | 50-54 |  |  |  |  |  |
| Switzerland | Females | low | 1922-1930 | 65-69 | 75-79 | 3 |  |  |  |  |  |  |  | 65-69 | 70-74 | 75-79 |
| Switzerland | Females | low | 1927-1935 | 60-64 | 75-79 | 4 |  |  |  |  |  |  | 60-64 | 65-69 | 70-74 | 75-79 |
| Switzerland | Females | low | 1932-1940 | 55-59 | 75-79 | 5 |  |  |  |  |  | 55-59 | 60-64 | 65-69 | 70-74 | 75-79 |
| Switzerland | Females | low | 1937-1945 | 50-54 | 70-74 | 5 |  |  |  |  | 50-54 | 55-59 | 60-64 | 65-69 | 70-74 |  |
| Switzerland | Females | low | 1942-1950 | 45-49 | 65-69 | 5 |  |  |  | 45-49 | 50-54 | 55-59 | 60-64 | 65-69 |  |  |
| Switzerland | Females | low | 1947-1955 | 40-44 | 60-64 | 5 |  |  | 40-44 | 45-49 | 50-54 | 55-59 | 60-64 |  |  |  |
| Switzerland | Females | low | 1952-1960 | 40-44 | 55-59 | 4 |  |  | 40-44 | 45-49 | 50-54 | 55-59 |  |  |  |  |
| Switzerland | Females | low | 1957-1965 | 40-44 | 50-54 | 3 |  |  | 40-44 | 45-49 | 50-54 |  |  |  |  |  |
| Switzerland | Females | high | 1922-1930 | 65-69 | 75-79 | 3 |  |  |  |  |  |  |  | 65-69 | 70-74 | 75-79 |
| Switzerland | Females | high | 1927-1935 | 60-64 | 75-79 | 4 |  |  |  |  |  |  | 60-64 | 65-69 | 70-74 | 75-79 |
| Switzerland | Females | high | 1932-1940 | 60-64 | 75-79 | 4 |  |  |  |  |  |  | 60-64 | 65-69 | 70-74 | 75-79 |
| Switzerland | Females | high | 1937-1945 | 55-59 | 70-74 | 4 |  |  |  |  |  | 55-59 | 60-64 | 65-69 | 70-74 |  |
| Switzerland | Females | high | 1942-1950 | 50-54 | 65-69 | 4 |  |  |  |  | 50-54 | 55-59 | 60-64 | 65-69 |  |  |
| Switzerland | Females | high | 1947-1955 | 45-49 | 60-64 | 4 |  |  |  | 45-49 | 50-54 | 55-59 | 60-64 |  |  |  |
| Switzerland | Females | high | 1952-1960 | 45-49 | 55-59 | 3 |  |  |  | 45-49 | 50-54 | 55-59 |  |  |  |  |
